# Endogenous protein adsorption and functional presentation at bone-regenerative biointerfaces

**DOI:** 10.3389/fbioe.2026.1965344

**Published:** 2026-10-15

**Authors:** Yunhe Zhang, Yin Xu, Chenhao Zhang, Baomin Lu

**Affiliations:** 1 Dalian Medical University, Dalian, China; 2 School of Stomatology, China Medical University, Shenyang, China; 3 Department of Orthopedics, Haicheng Orthopedics Hospital, Anshan, China; 4 Department of Trauma and Joint Surgery, People’s Hospital of China Medical University (The People’s Hospital of Liaoning Province), Shenyang, China

**Keywords:** biointerface, biomaterials, bone regeneration, fibronectin, functional protein presentation, osteoimmunology, protein adsorption, protein corona

## Abstract

Bone-regenerative materials rapidly acquire proteins from blood, marrow fluid, and the surrounding tissue. The resulting interface depends on protein quantity, identity, orientation, conformation, and exchange, rather than total adsorption alone. This narrative critical review examines selected mechanisms through which these features influence receptor recognition and responses relevant to bone repair. We use the descriptive term interfacial protein state and propose a joint reporting profile for comparisons made under matched exposure and sampling conditions. Primary evidence is distinguished by protein source, experimental setting, causal perturbation, and limits of interpretation. The strongest mechanistic examples concern fibronectin presentation and integrin signaling, selected fibrinogen-dependent inflammatory pathways, and material-assisted presentation of exogenous growth factors. Quantitative examples of surface density, accessibility, dissociation, and retained BMP-2 dose are reported with their experimental conditions. Autologous platelet-rich plasma and platelet-rich fibrin are considered as clinically relevant precoatings whose preparation and release behavior require separate characterization. Competition between host cells and bacteria for the conditioned interface is also addressed. Hemostatic-inflammatory, angiogenic-matrix-forming, and mineralization-remodeling processes are presented as overlapping biological contexts, not as a validated schedule for programming protein exchange. Evidence from purified coatings and dispersed nanoparticles is not treated as proof of endogenous recruitment or clinical bone healing. Predicting outcomes across materials, guiding patient-specific implant selection, and replacing growth-factor delivery through endogenous capture remain unvalidated. The proposed measurements organize causal tests and identify evidence gaps; they do not define a universal protein-state score or an established clinical design rule.

## 1 Introduction: from material surfaces to acquired biological identity

Critical-sized defects, osteoporotic fractures, infected defects, and peri-implant bone loss still present difficult clinical problems. Porous metals, bioceramics, hydrogels, nanoparticles, and additively manufactured scaffolds offer considerable control over shape, mechanics, and local delivery, but these nominal properties are only the starting point. Once a device is implanted, plasma, marrow-fluid, and interstitial-fluid proteins reach the surface within seconds to minutes and compete for available sites. Fast, abundant species usually dominate the earliest contact. They do not necessarily stay there. Proteins with stronger interfacial interactions can displace part of the first layer, while tightly and loosely associated fractions coexist and continue to exchange. The Vroman-like process is therefore a moving, multilayered one rather than a sequence of complete replacements (Cedervall et al., 2007; Lundqvist et al., 2008; Monopoli et al., 2012; Tenzer et al., 2013; Walkey and Chan, 2012; Lynch and Dawson, 2008; Nel et al., 2009; Monopoli et al., 2011; Noh and Vogler, 2007). By the time immune cells, endothelial cells, osteoprogenitors, or osteoblasts arrive, they are usually contacting a material-protein composite, not bare titanium, hydroxyapatite, or polymer.

That sequence changes the experimental question. Hydrophilicity, roughness, and total adsorbed mass can describe a surface or its overall protein load, but they do not tell us what a receptor can actually bind. The useful measurements are more specific: which proteins persist, which molecular domains point outward, whether receptor-binding sites remain accessible, and how quickly each population is exchanged. Structural change also needs a functional readout. A protein that extends reversibly and exposes a normal binding domain is biologically different from one that unfolds irreversibly and presents an inflammatory epitope. For a bone-regenerative interface, the difficult part is to support the early inflammatory response, vascular growth, and osteogenesis without pushing coagulation or complement into a damaging range.

In this review, anchoring is not restricted to covalent attachment. We include selective capture, retention, and organization of host proteins produced by physicochemical interactions with a complex biofluid. We use conformational reprogramming more narrowly. The term refers to a material-induced change in protein conformation, orientation, assembly, or epitope accessibility only when that change alters receptor engagement relative to the soluble state. Not every change in secondary structure meets that standard. Limited extension can preserve or uncover a physiological function; deeper denaturation may instead expose nonphysiological epitopes, promote aggregation, trigger complement or scavenger receptors, and speed clearance (Corbo et al., 2016a; Corbo et al., 2016b; Deng et al., 2011; Schöttler et al., 2016).

The argument is not that a useful implant should adsorb more protein. Nor do we expect one ‘best’ corona to work across different materials and patients. We focus instead on the functional interfacial protein state: the combination of protein identity and abundance with orientation, conformation, assembly, functional-epitope accessibility, and exchange kinetics. Any one of these variables can change receptor recognition while the others appear similar. A mechanistic experiment should therefore separate four questions. Which interfacial protein state was formed? How long did it persist? Did the immune, vascular, or osteogenic response depend on that state? And was the state still present after manufacturing and sterilization? Framing the work this way also makes it easier to distinguish a protein-mediated effect from ion release, mechanics, direct membrane interactions, or another material property.

## 2 Literature identification and review scope

We conducted this work as a mechanism-oriented narrative critical review rather than a quantitative synthesis. Searches covered PubMed/MEDLINE, relevant publisher databases, backward citation tracking, and official regulatory sources. The main search ran from database inception to 26 July 2026; we then added a focused update through 10 August 2026 for newly published studies directly related to the mechanism. Our purpose was to map causal evidence and translational limits, not to calculate a pooled effect size. During revision, a targeted literature check on 26 September 2026 added primary studies on quantitative FN adsorption, blood-product precoatings, and bacterial use of host proteins; it was not an exhaustive update of all review topics.

Representative search blocks combined (“protein corona” OR “protein adsorption” OR “conditioning film” OR “acquired biological identity”) with (bone OR osseointegration OR implant* OR scaffold* OR titanium OR hydroxyapatite), and were expanded with terms for conformation, orientation, epitope accessibility, fibronectin, fibrinogen, complement, immunoglobulin, platelet, neutrophil, osteopontin, fetuin-A, growth-factor presentation, proteomics, single-cell or spatial transcriptomics, and patient-specific biofluids. Related searches addressed sterilization, biocompatibility, coagulation, and regulatory translation.

We prioritized English-language original studies that linked a defined material property to a measured protein feature—identity, orientation, conformation, assembly, exchange, or receptor accessibility—and then reported a receptor, cellular, vascular, immune, osteogenic, or *in vivo* bone outcome. Reviews were used to map broader areas, but central mechanistic claims were traced back to primary studies when possible. Studies that measured only total adsorption were retained as context. They were not taken as evidence that a specific protein-state mechanism had been demonstrated.

We separated the evidence into three categories before interpreting the literature. Established findings required direct primary data and convergence across independent studies or platforms. Mechanistic inferences had a coherent biological basis and some causal support, but still lacked a necessity, rescue, cross-scale, or indication-matched validation step. Testable hypotheses were prospective design or clinical propositions without prospective validation. This distinction is especially important when evidence is transferred between nanoparticles, macroscopic implants, porous scaffolds, hydrogels, and mineral interfaces; these systems were compared, not treated as interchangeable.

We also labelled the experimental domain of major claims so that evidence from different scales did not appear equivalent. IV refers to purified-protein, defined-mixture, or model-surface *in vitro* work; EX to *ex vivo* human plasma, marrow fluid, wound fluid, or other complex human biofluids; AN to non-bone animal evidence; BA to bone-or implant-relevant animal evidence; HU to human tissue, *in vivo* exposure, or observational evidence; and CL to evidence prospectively linked to a clinical bone-healing or implant outcome. A mechanism supported only by IV, AN, or nanoparticle studies is not described here as clinically established without cross-scale validation.

This is a narrative review, so the search should not be read as a systematic-review workflow. We did not use exhaustive retrieval, duplicate screening, formal risk-of-bias scoring, or PRISMA reporting. Instead, we make the boundary explicit by reporting the search logic, the evidence categories, and a representative set of primary studies. The studies used to support the principal causal claims are listed in Table 1.

**TABLE 1:** Representative primary evidence, protein sources, and limits of inference.

Study and domain	Protein source/interface	Perturbation or measurement	What the study supports	Main evidence boundary
Tang and Eaton, 1993; Tang et al., 1996 [IV/AN]	Host fibrin (ogen), defined protein conditions; generic biomaterials	Fibrin (ogen)-linked leukocyte recruitment; inflammatory sequence and Mac-1-related tests	A fibrinogen-dependent inflammatory mechanism	Not bone-specific; materials and implantation sites vary
Keselowsky et al., 2003, 2004 [IV]	Purified FN on model surface chemistries	Integrin-selective adhesion and focal-adhesion signaling	Surface chemistry can alter functional FN presentation	Reductionist coatings; not endogenous recruitment or clinical bone healing
Llopis-Hernández et al., 2013 [IV]	Purified FN on PEA and PMA.	Surface density, AFM organization, HFN7.1 accessibility and cell remodeling	Similar FN mass can coexist with different assembly/accessibility	No matched exchange half-life; accessibility does not isolate orientation
Lüthen et al., 2005; Att et al., 2009 [IV/BA]	Titanium exposed to culture or host proteins; cell-associated FN organization	Roughness or surface aging; osteoblast adhesion and osteoconductivity	Processing-dependent biological behavior of titanium	The protein-mediated contribution is incompletely isolated
Deng et al., 2011 [IV/AN]	Adsorbed fibrinogen on nanoparticles	Protein unfolding, Mac-1 activation and inflammatory response	A conformation–receptor–inflammation link	Nanoparticle results do not transfer unchanged to bulk implants
Llopis-Hernández et al., 2016; Xiao et al., 2022 [IV/BA]	Defined FN or FN/VN coatings with exogenous recombinant BMP-2	Assembly, domain/receptor interactions and osteogenic outcomes	Material-assisted growth-factor presentation at low experimental dose	Does not quantify endogenous BMP-2 capture or establish clinical dose equivalence
Alfarsi et al., 2015; Park et al., 2018 [IV]	Platelets/blood-derived components at titanium interfaces	Surface changes, platelet responses and releasate effects on macrophages	Early blood–material events can change immune signaling	Early *in vitro* endpoints; long-term bone necessity unproven
Araújo-Gomes et al., 2019 [IV]	Adsorbed complement proteins on biomaterial surfaces	Surface-associated proteins and macrophage polarization assays	Complement-associated modulation of macrophage behavior	Donor, surface and assay context limit generalization
Fetz et al., 2021 [IV]	Adsorbed IgG on electrospun polymer fibers	FcγRIIIb blockade and NET release	A protein–receptor–neutrophil mechanism	Not a bone implant model
Kuyl et al., 2021 [BA]	Host-derived NETs in a murine tibial implant setting	PAD4 inhibition or DNase-mediated clearance	NET accumulation contributes to fibrotic osseointegration failure	Does not alone identify a specific adsorbed protein state; clinical efficacy unproven
Zhu et al., 2017, 2019 [IV/BA]	Biofluid-derived corona on magnetic hydroxyapatite scaffolds	Corona characterization, pathway analysis and osteoimmune outcomes	Scaffold-associated proteins are linked to regeneration-related responses	Magnetic, ionic and corona effects remain partly confounded
Kolar et al., 2011; Hoff et al., 2016 [HU]	Native human fracture hematoma	Human tissue and early wound-milieu characterization	A clinically relevant source environment for interface formation	Does not test a material-induced protein state
Hadjidemetriou et al., 2015, 2019 [AN/HU]	Circulating host proteins on recovered liposomes	Authentic *in vivo* exposure and human proof of concept	*In vivo* coronas can differ from simplified *ex vivo* conditions	Nanomedicine context; orthopedic relevance is inferential
Mao et al., 2026 [IV/BA]	Disease-associated corona on Ti and ZrO_2_ wear particles	Proteomics, macrophage signaling, osteoclast responses and bone destruction	Diabetes-associated changes in particle biological identity and osteoimmune effects	Wear-particle osteolysis; not a regenerative scaffold or prospective selection study
Kobayashi et al., 2016 [EX]	Donor-derived PRP, PRF and A-PRF.	Preparation comparison and growth-factor release up to 10 days	Preparation-dependent release behavior	Six donors; release is not proof of clinical efficacy or interface mechanism
Fernandes et al., 2022 [CL]	Liquid autologous PRF applied to dental implants	Randomized split-mouth trial; 15 patients, 30 implants, 1-year follow-up	No statistically significant between-group differences in assessed outcomes	Protocol-specific clinical result; no proof of equivalence or protein-state causality
Sinha et al., 2000; McDevitt et al., 1994 [IV]	FN-or fibrinogen-mediated bacterial interactions	Heterologous FN-binding protein expression; clumping-factor characterization	Staphylococci can exploit host adhesion proteins	Mechanistic microbial evidence; not a bone-healing or clinical coating trial
Damiati et al., 2022 [IV]	FN/BMP-2 presentation on coated titanium nanotopography	MSCs and *P. aeruginosa* studied in a combined material system	Host-cell support and biofilm inhibition can be examined together	Composite design; organism-specific evidence, not clinical infection prevention

Domains are defined in Section 2: IV, defined *in vitro* systems; EX, *ex vivo* human biofluids/blood products; AN, non-bone animal evidence; BA, bone/implant-relevant animal evidence; HU, human tissue or observational exposure; CL, prospective clinical outcomes. Domain labels identify setting, not quality or causal strength.

This review evaluates selected mechanisms by which protein adsorption and functional presentation can influence responses relevant to bone regeneration. The framework is intended to organize measurements and causal tests. Its ability to predict bone healing across materials, indications, or patients remains unvalidated. Evidence obtained with purified coatings, recombinant growth factors, or dispersed nanoparticles is identified separately from endogenous recruitment at a bone-contacting implant.

## 3 Conceptual framework: components and measurable dimensions of the interfacial protein state

Four components are separated in our framework. The first is the source proteome. Plasma, serum, marrow aspirate, fracture hematoma, interstitial fluid, and cell-secreted matrix do not supply the same proteins or proteoforms. Serum preparation, for example, depletes or changes fibrinogen and several coagulation factors, whereas marrow fluid contributes local matrix fragments, lipids, immunoglobulins, hematopoietic proteins, and skeletal regulators. A fracture hematoma adds another combination: platelet products, complement fragments, proteases, damage-associated molecules, hypoxia, and a cell population that changes with time (Kolar et al., 2011; Hoff et al., 2016). The second component is the material interaction field, set by chemistry, surface energy, charge heterogeneity, curvature, nanoscale confinement, ion release, and deformation. The third is the acquired interfacial protein state itself, described by identity, stoichiometry, orientation, conformation, assembly, post-translational state, and residence time. The fourth is cellular reading of that state through integrins, complement and Fc receptors, scavenger and pattern-recognition receptors, growth-factor receptors, and glycocalyx-associated molecules. Cells then modify what they read. Traction, protease release, and new matrix deposition feed back on the interface, so the process is bidirectional (Cedervall et al., 2007; Monopoli et al., 2012; Keselowsky et al., 2003; Bai et al., 2017; García, 2006).

Five dimensions make the interfacial protein state experimentally tractable: quantity, identity, orientation, conformation, and dynamics. They describe related features, but one cannot substitute for another. More adsorbed FN, for example, does not establish that its RGD/PHSRN region is accessible or that an integrin-dependent response follows. The term is descriptive: it refers to the measured protein profile at a specified interface and time. Assembly is considered with conformation, while displacement, rearrangement, and degradation are considered with dynamics. Epitope accessibility is a joint proxy affected by orientation, conformation, and crowding; it does not independently identify all three.

For bone-regenerative applications, endogenous proteins can be provisionally organized into four functional groups. Adhesive ECM proteins, dominated by FN, vitronectin, fibrin, collagen-derived fragments, and osteopontin, connect cells to the interface and organize cytoskeletal force. Hemostatic and immune proteins, including fibrinogen, complement, immunoglobulins, apolipoproteins, and acute-phase proteins, regulate clotting, opsonization, recruitment, and foreign-body recognition. Carrier and homeostatic proteins, such as albumin, transferrin, and fetuin-A, influence passivation, ion transport, mineralization, and nanoparticle uptake. Finally, morphogen-binding proteins and ECM assemblies capture, protect, and spatially present BMPs, VEGF, FGFs, TGF-β-family ligands, and chemokines. This classification is functional, not exclusive. FN can simultaneously support adhesion, growth-factor presentation, and immune-cell migration; fibrin can be both a clotting product and a regenerative matrix; and fetuin-A can inhibit ectopic mineral precipitation while participating in the corona of mineral-based scaffolds (Cedervall et al., 2007; Monopoli et al., 2012; Keselowsky et al., 2003; Bai et al., 2017; García, 2006).

Endogenous anchoring also differs from three commonly used approaches. Direct immobilization attaches a purified protein to the material before implantation. Peptide functionalization reproduces a limited motif such as RGD. Cell-membrane coating transfers a preassembled biological shell. Endogenous anchoring leaves final interface formation to the host biofluid but biases which proteins are captured and how they are displayed. Reducing reliance on recombinant-factor dosing through endogenous capture is an intended benefit that still requires direct demonstration; the interface may also change with healing. The trade-off is loss of control: donors differ, proteins compete, conformational outcomes are uncertain, and a disease-altered proteome may build an unintended biological identity. We do not regard endogenous anchoring as intrinsically safer or more physiological. It is useful only if the resulting state is predictable in the population for which the material is intended (Cedervall et al., 2007; Monopoli et al., 2012; Keselowsky et al., 2003; Bai et al., 2017; García, 2006).

Interface terminology should remain scale specific. For dispersed nanoparticles, protein corona describes a particle-associated layer that can govern colloidal behavior, transport, uptake, and intracellular fate. Macroscopic implants and porous scaffolds instead develop a conditioning film and provisional matrix containing proteins, lipids, ions, platelets, extracellular vesicles, and subsequently cell-deposited ECM. In this review, interfacial protein state refers specifically to the measurable protein identity, structure, accessibility, and dynamics within these broader interphases; evidence from nanoparticles is not extrapolated to bulk implants without an explicit scale qualification (Monopoli et al., 2012; Barberi and Spriano, 2021; Bai et al., 2021).

### 3.1 Anchored quantity is necessary but insufficient

Total protein mass answers a capacity question, not a function question. Two surfaces can carry the same mass but a different protein repertoire. Even for one protein, equal loading can produce different biological effects if folding, orientation, or neighboring molecules differ. FN is a clear example. Increasing the amount of adsorbed FN helps osteogenic signaling only when the RGD motif and PHSRN synergy site remain exposed in a geometry that permits integrin binding. If either region is turned toward the material or sterically blocked, adding more FN does not restore the missing signal (Keselowsky et al., 2003; Michael et al., 2003; Keselowsky et al., 2004).

A defined-protein experiment provides a quantitative anchor rather than a universal target density. Adsorption from 2 or 20 μg/mL FN solutions for 10 min produced approximately 47 or 340 ng/cm^2^, respectively, on both poly (ethyl acrylate) (PEA) and poly (methyl acrylate) (PMA) (Llopis-Hernández et al., 2013). The values refer to those materials and exposure conditions, and the study quantified adsorption indirectly from the nonadsorbed fraction. Equal mass did not imply equal FN assembly or site accessibility. A matched comparison is developed in Section 3.6; Table 2 collects the quantitative examples and their limits.

**TABLE 2:** Quantitative anchors and assay-specific interpretations.

Readout	System and conditions	Numerical or operational anchor	Interpretation/limitation	Source
FN surface density	Purified FN on PEA and PMA; 10-min adsorption	2 and 20 μg/mL coating solutions gave approximately 47 and 340 ng/cm^2^, respectively	Comparable mass on both polymers; condition-specific values, not optimal targets	Llopis-Hernández et al., 2013
FN accessibility	HFN7.1 antibody assay on the same PEA/PMA comparison	At 20 μg/mL coating, accessibility normalized to FN amount differed between surfaces	Relative assay signal; no absolute exposed-site percentage without calibration	Llopis-Hernández et al., 2013; Section 8.2
Initial corona formation	Plasma-exposed nanoparticles	Complex corona detected within <0.5 min	Formation onset is distinct from exchange or residence time	Tenzer et al., 2013
HSA dissociation	200-Nm 85:15 NIPAM/BAM particles	Dissociation rate approximately 4 × 10^−4^ s^-1^; half-life approximately 30 min	Protein/material-specific dissociation; not whole-corona Vroman replacement	Cedervall et al., 2007
Sequential competition	HSA on octyl sepharose, then challenge with an IgG-containing solution	1-h Initial adsorption followed by 1-h challenge	Protocol contact times; no exchange half-life inferred	Noh and Vogler, 2007
BMP-2 mass/dose	Recombinant BMP-2 on polymer/FN-coated sleeves; 2.5-mm mouse radial defect	Approximately 15 ng per implant; surface density 100 ± 8 ng/cm^2^	Exogenous retained dose; not endogenous capture or clinical dose equivalence	Llopis-Hernández et al., 2016
BMP-2 loading solution	*In vitro* adsorption protocol in the FN-assisted presentation study	25 ng/mL BMP-2 adsorption solution	Solution concentration must remain distinct from retained mass and implant dose	Llopis-Hernández et al., 2016
Endogenous BMP-2 activity	Patient-fluid recruitment: Proposed validation protocol	Recovery-controlled retained mass plus a matched soluble dose-response and neutralization/blockade	No universal captured activity equivalent is established by the cited loading studies	Section 5.4; proposed measurement requirement

Approximate values retain the source study’s context. No universal FN, density, Vroman exchange timescale, accessibility threshold, or endogenous BMP-2, therapeutic dose is inferred. Formation, dissociation, displacement, and release are distinct processes.

### 3.2 Which proteins occupy the interface, and where

Early composition is set by more than equilibrium affinity. Transport to the surface, protein abundance, available area, and lateral protein-protein interactions all contribute. A rapidly diffusing, abundant protein can therefore dominate the first layer even when its final affinity is modest. Later, a less abundant species may replace part of that layer because it makes more surface contacts, or a protein complex may be stabilized by interactions with neighboring adsorbates. The classical Vroman description captures part of this sequence, but actual coronas can also grow by multilayer formation, transient complexes, cooperative binding, enzymatic cleavage, and cell-mediated deposition (Cedervall et al., 2007; Lundqvist et al., 2008; Noh and Vogler, 2007). A high-affinity late arrival does not have to erase what came first. It may fill gaps, bind an already adsorbed protein, or accumulate in the outer layer. As a result, the protein most visible to a receptor may be neither the most abundant nor the most tightly bound species.

The denominator used for adsorption is also scale dependent. A nanoporous or fibrous scaffold can have a large geometric area that many proteins cannot physically reach, and roughness changes both hydration and local transport. For nanoparticles, curvature sets how many contact points a protein can make and how strongly neighboring proteins crowd it. At high surface density, proteins can change conformation because they collide with one another even when their direct interaction with the material is weak. Normalizing adsorption only to projected area can therefore create an apparent structure-function relation that disappears once accessible area, pore connectivity, and protein-size distribution are considered (Cedervall et al., 2007; Monopoli et al., 2012; Keselowsky et al., 2003; Bai et al., 2017; García, 2006).

Hard and soft corona are useful experimental labels, but they are not a complete map of the interface. A tightly bound species can serve as a base for later assembly, whereas a weak outer species may be the one a cell receptor encounters first. This distinction is sensitive to sample preparation. Strong washing before proteomics removes many weak components and can turn several spatial layers into one averaged composition. Protein identity is therefore more useful when it is reported together with receptor accessibility, exposure time, isolation conditions, and the biofluid used to form the layer.

### 3.3 Orientation changes receptor access without changing protein identity

Orientation can change function even when protein identity and abundance do not change. Receptor-binding regions that face the fluid remain available to cells; the same regions are effectively silent when they lie against the material. This issue applies to the integrin-binding domains of FN, vitronectin, and osteopontin, to the Fc region of immunoglobulins, and to receptor-recognition sites in complement fragments. Charge patterning, ligand spacing, curvature, and directional coordination can therefore be more useful for controlling orientation than simply increasing surface loading.

### 3.4 Conformation: useful exposure versus denaturation

Conformation has to be read at more than one scale. Secondary-structure measurements report the balance among α-helix, β-sheet, turns, and disorder. Domain-level measurements ask whether modular regions stay packed or extend. A third level is functional presentation: is the receptor-or growth-factor-binding site actually exposed in a usable geometry? These measurements are related, but one cannot stand in for another. More β-turn content, for example, may accompany better adhesion without proving that the RGD loop is exposed or that PHSRN is positioned correctly for α5β1 binding. The reverse problem also occurs. Most secondary structure can remain intact while rotation places the critical domain against the surface. Global spectroscopy alone therefore cannot establish site-specific function (Cedervall et al., 2007; Monopoli et al., 2012; Keselowsky et al., 2003; Bai et al., 2017; García, 2006).

There is no single amount of unfolding that separates a useful state from a damaged one. The boundary depends on the protein and on what the interface is expected to do. Partial FN extension can resemble aspects of cell-driven fibrillogenesis and uncover cryptic binding regions. A similar degree of structural change in fibrinogen can instead reveal inflammatory epitopes. One interface may also trade one function for another: a state that improves osteoblast attachment can at the same time increase platelet adhesion or bacterial binding. We describe a conformational change as functionally beneficial only when a measured structural change is linked to a beneficial receptor-dependent phenotype and has been distinguished experimentally from aggregation or loss of native function (Cedervall et al., 2007; Monopoli et al., 2012; Keselowsky et al., 2003; Bai et al., 2017; García, 2006).

FN shows the potentially useful side of this process. After adsorption it can move from a compact form toward an extended or fibrillar organization, making integrin-and growth-factor-binding regions more accessible. Fibrinogen illustrates the opposite direction: too much unfolding can uncover motifs recognized by Mac-1. Albumin is not exempt; when strongly denatured, it can gain affinity for scavenger receptors (Deng et al., 2011). Taken together, these examples do not support maximizing unfolding. The design target is a limited conformational range that preserves the desired function without creating a new injury signal.

### 3.5 Protein exchange and cell-driven remodeling

The adsorbed layer is not chemically or mechanically frozen after implantation. Flow, ionic strength, pH, oxidative stress, proteases, and newly deposited matrix keep changing it. The provisional clot that dominates the first hours is not the same environment encountered several days later, when marrow-derived and granulation-tissue proteins become more prominent. Cells add another source of change: they pull on FN, deposit fresh ECM, and remove early proteins by proteolysis. It is therefore more realistic to treat a regenerative surface as a protein niche that evolves with healing rather than as a coating established once at implantation.

Initial corona formation, protein dissociation, and competitive displacement require different measurements. A complex plasma corona formed within less than 0.5 min in the nanoparticle systems studied by Tenzer et al. (2013). Cedervall et al. (2007) estimated an HSA dissociation rate of approximately 4 × 10^−4^ s^-1^, corresponding to a half-life of approximately 30 min, for a 200-nm 85:15 N-isopropylacrylamide/N-tert-butylacrylamide (NIPAM/BAM) particle system. This is a protein-and material-specific dissociation result, not a general Vroman timescale. In a sequential competition experiment, Noh and Vogler (2007) used 1 h of HSA adsorption on octyl sepharose followed by a 1-h challenge with an IgG-containing solution. Those are experimental contact times, not a measured exchange half-life. Competitive exchange should instead be followed as component-specific surface density versus time under a defined challenge, with flow, temperature, protein concentrations, and fitting assumptions reported.

We also use different verbs for different levels of evidence. ‘Mediates,’ ‘requires,’ and ‘is necessary’ are reserved for studies that directly perturb the proposed mechanism through depletion, receptor blockade or knockdown, rescue, or an equivalent test. ‘Supports’ is used when evidence converges but a causal step is still missing. ‘May,’ ‘could,’ and ‘we propose’ mark inference or hypothesis. The arrows in Figures 1, 2 should therefore not be interpreted as carrying the same evidentiary weight.

**FIGURE 1:**
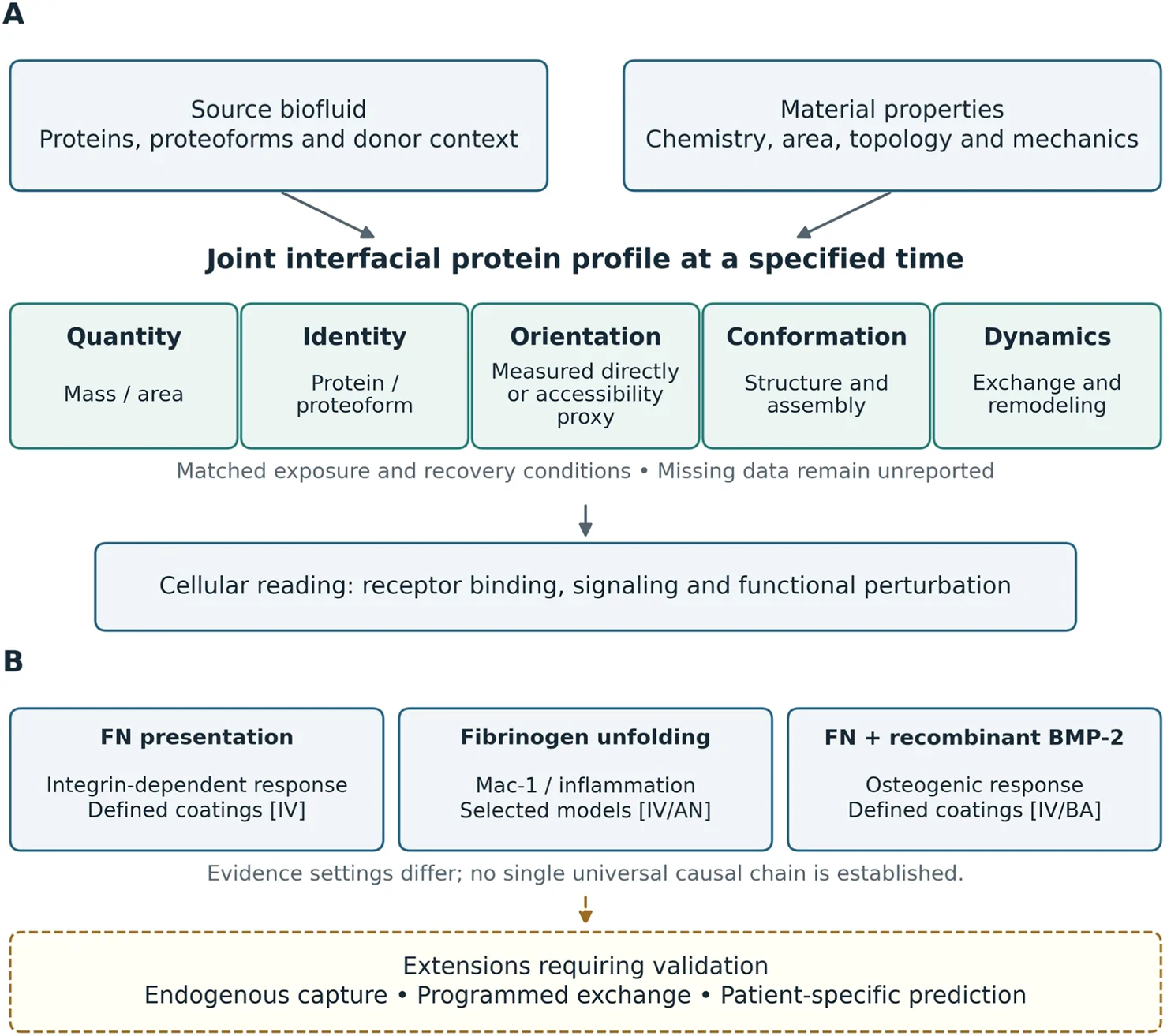
Measured protein states and boundaries of causal inference. The source biofluid and material properties jointly shape the acquired protein interface. Quantity, identity, orientation/accessibility, conformation/assembly, and dynamics are reported together, while receptor-dependent function is tested separately. Solid arrows organize measured relationships in selected experimental systems; they do not assert that every step has been demonstrated in one bone-healing model. Examples include FN presentation and integrin signaling in defined systems, fibrinogen–Mac-1 inflammation, and exogenous BMP-2 presentation in a bone-relevant animal model. Dashed arrows indicate proposed extensions to endogenous capture, programmable exchange, and patient-specific prediction. Missing dimensions remain unmeasured rather than being inferred from downstream outcomes. See Sections 2 and 3 and Table 1 for study domains and limitations. **(A)** Components and joint measurements **(B)** Selected experimentally supported links.

**FIGURE 2:**
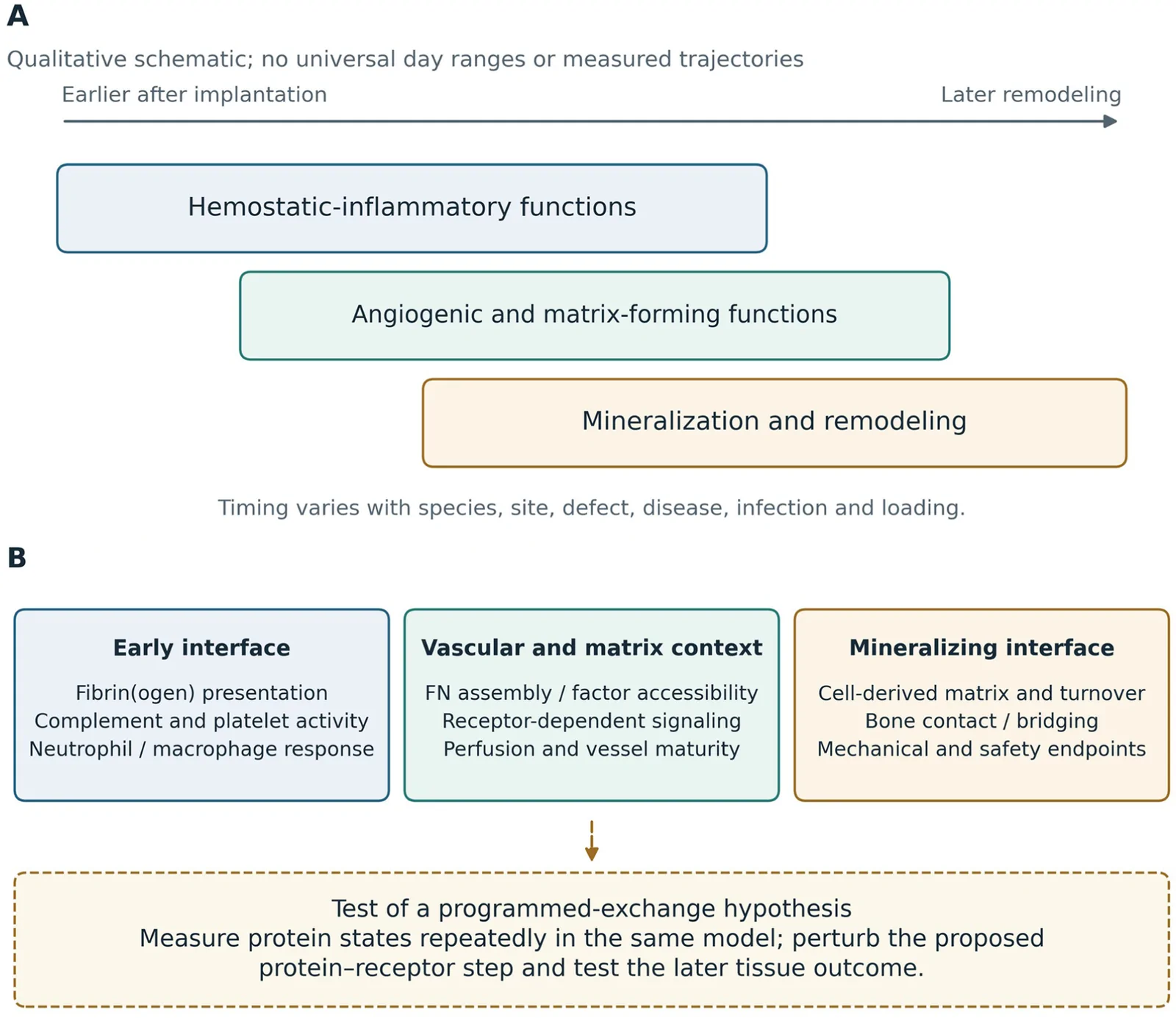
Overlapping biological windows and measurements for testing temporal hypotheses. The bands are a qualitative organizing schematic, not measured curves, fixed day ranges, or a validated repair schedule. Hemostatic-inflammatory, angiogenic-matrix-forming, and mineralization-remodeling functions overlap and vary with species, site, defect, infection, systemic disease, and loading. Suggested observations connect protein-state measurements to contemporaneous cellular and tissue endpoints. Direct tests of programmed endogenous exchange would require repeated measurements and causal perturbations within the same model. The diagram does not establish that one protein sequence is necessary or sufficient for bone healing. See Section 4. **(A)** Overlapping biological windows **(B)** Pair interface measurements with contemporaneous outcomes.

Figure 1 separates the source biofluid, material properties, joint protein-state measurements, and receptor-dependent responses. It distinguishes selected experimentally supported links from extensions that still require validation.

Table 3 specifies observables, units or assay-specific readouts, and limitations for the five dimensions. The joint comparison and its use in a same-study example are described in Section 3.6.

**TABLE 3:** Operational profile for comparing interfacial protein states.

Dimension	Observable and reporting unit	Comparison under matched conditions	Principal limitation
Quantity	Retained mass (ng/cm^2^), molecules per particle, or mass per implant; state the area denominator	Direct or recovery-controlled quantification; QCM-D or SPR with the fitted model and appropriate controls	Hydrated QCM-D mass differs from dry protein mass; relative abundance is not absolute capture
Identity	Protein/proteoform identity and absolute abundance where calibrated; otherwise relative fraction or enrichment	LC-MS/MS with fluid, process-blank, recovery, and donor controls; distinguish intact protein from fragments	A detected peptide does not establish intact, receptor-active protein
Orientation/accessibility proxy	Orientation from validated domain-specific labels; otherwise normalized antibody or receptor-binding signal in assay-specific units	State label position, antibody conditions, background subtraction, and normalization to retained protein	Accessibility jointly reflects orientation, conformation, and crowding; it is not a direct orientation angle
Conformation/assembly	Secondary-structure fraction (% where supported), domain exposure, molecular extension (nm), or fibril organization	Pair spectroscopy, AFM, HDX-MS, or limited proteolysis with an orthogonal site/function assay	Global structure does not prove functional epitope exposure; recovery can alter structure
Dynamics	Component-specific density versus time; fitted dissociation rate (s^-1^) or half-life (min) when justified	Define flow, challenge proteins, concentration, temperature, wash conditions, and kinetic model	Contact time is not an exchange half-life; single-component dissociation is not whole-corona replacement

Report the five dimensions together for each material, fluid, and time point. Keep raw values and uncertainty; mark unmeasured dimensions as not reported. Receptor-dependent function is a separate validation outcome. No universal weighted score or threshold is proposed (Section 3.6).

### 3.6 Comparing interfaces using a joint reporting profile

For a specified material, biofluid, and sampling time, the interfacial protein state is reported as a joint profile of quantity, identity, orientation or explicitly identified accessibility proxies, conformation, and dynamics. These dimensions are compared under matched conditions and remain separate measurements. Unmeasured dimensions are recorded as not reported. Receptor-dependent activity is used to test the functional significance of a profile difference; it is not used to fill in missing structural measurements. We do not assign a universal composite score because clinically relevant weights and thresholds have not been validated.

A comparison should use the same donor or donor-matched fluid, protein concentration, temperature, contact time, flow, and recovery or washing procedure. Quantity should be normalized to a stated area measure, with accessible area distinguished from projected area. Each dimension should retain its raw readout, assay controls, between-interface effect estimate, and uncertainty. At several time points, the comparison is repeated as a series of profiles rather than reduced to one endpoint. Evidence from different fluids or studies should not be combined into a nominally complete profile for one interface.

For example, PEA and PMA exposed to 20 μg/mL purified FN for 10 min had similar FN surface densities of approximately 340 ng/cm^2^ (Llopis-Hernández et al., 2013). Identity was controlled by using purified FN. AFM distinguished fibrillar assembly on PEA from a more globular organization on PMA, and HFN7.1 binding normalized to adsorbed FN distinguished accessibility at the higher coating concentration. This antibody signal is an accessibility proxy; orientation was not independently resolved. An exchange half-life was not reported for this matched comparison. Thus, the quantity and identity dimensions were similar, conformation/assembly and accessibility differed, and orientation and exchange kinetics remained incompletely measured. The profile preserves those limits rather than borrowing a half-life from another material.

### 3.7 Autologous PRP and PRF precoatings

Autologous blood-product precoatings provide a clinically relevant test case for the proposed measurements. Platelet-rich plasma (PRP) and platelet-rich fibrin (PRF) supply patient-derived components through an *ex vivo* preparation step, which differs from selective recruitment after implantation. Centrifugation, anticoagulation, activation, platelet and leukocyte content, and fibrin organization affect the applied preparation. A characterization should therefore report the preparation protocol and donor context, the amount retained per implant or accessible area, clot or fibrin structure, and release or loss after exposure to the relevant fluid. Subsequent adsorption from host fluid can further alter that initial layer.

Preparation-dependent release is experimentally demonstrable, but it does not itself establish clinical benefit. Kobayashi et al. (2016) compared PRP, PRF, and advanced PRF in samples from six donors over observations extending to 10 days and found different growth-factor release profiles. In a randomized split-mouth trial involving 15 patients and 30 implants, Fernandes et al. (2022) found no statistically significant between-group differences in the assessed outcomes after liquid-PRF coating. This result does not establish equivalence or exclude benefits under other protocols; it limits an unqualified efficacy claim. Neither growth-factor content nor clinical application alone validates a particular protein-state mechanism. The evidence map in Table 1 therefore separates blood-product release experiments from implant-outcome evidence.

## 4 Temporal context: overlapping protein-interface functions during repair

Figure 2 organizes the discussion into overlapping biological windows, without assigning universal day ranges or a validated sequence of material-controlled protein states. Hemostasis and innate immune activation overlap with fibrin remodeling, endothelial sprouting, and osteogenic commitment. Species, anatomical site, defect size, infection, systemic disease, and loading can shift their timing. The following sections provide biological context for choosing observations and endpoints; they do not demonstrate that changing endogenous protein exchange alone coordinates all stages of bone repair. Deliberately programming such a sequence remains a hypothesis requiring time-resolved protein measurements and causal perturbation in the same model (Xie et al., 2020; Wen et al., 2023; Wan et al., 2024).

Time averaging is not the only source of error; spatial averaging can be equally misleading. Local surface energy, ion release, and curvature shape adsorption at the material itself, but fibrin and inflammatory-cell secretions may dominate only a few micrometers away. Conditions can also differ across the defect. The center may be hypoxic, acidic, nutrient poor, and rich in proteases, whereas the host-bone edge contains collagen fragments, mineral ions, and osteogenic cells. A single scaffold can therefore support several protein states at once. Bulk recovery and pooled proteomics erase that local variation. This provides one plausible explanation for a material that shows a favorable mean corona while developing fibrous encapsulation in one region and direct bone contact in another (Xie et al., 2020; Wen et al., 2023; Wan et al., 2024; Abedi et al., 2025).

### 4.1 Early hemostatic and inflammatory interface

Platelets are among the first cells to read the protein-coated surface. Their adhesion and activation are influenced by adsorbed fibrinogen, von Willebrand factor, FN, and complement. Surface context matters: titanium nanotopography and calcium chemistry can change early platelet and macrophage responses, and releasates from platelets exposed to different titanium surfaces can alter macrophage inflammatory gene expression (Alfarsi et al., 2015; Park et al., 2018). Activated platelets then release PDGF, TGF-β, chemokines, and extracellular vesicles that recruit leukocytes and progenitors. This response has a useful range, not a single desirable maximum. An organized provisional matrix can localize signals and provide traction, while excessive platelet activation increases thrombogenic and neutrophil-recruiting signals.

Neutrophils act on the interface with proteases, reactive oxygen species, and extracellular traps before many later osteogenic readouts are collected. Protein adsorption on a biomaterial can change receptor-specific NET release. In a murine tibial implant model, reducing or clearing PAD4-dependent NETs decreased peri-implant fibrosis (Fetz et al., 2021; Kuyl et al., 2021). This creates a timing problem for interpretation. If an osteogenesis assay begins after neutrophil activity has already peaked, early inflammatory damage may be invisible even though it was triggered by the initial protein layer. A surface can therefore look favorable in a later cell assay while having produced a damaging early response.

Macrophages do not respond only to soluble cytokines. They also read mechanical and conformational information in the adsorbed layer through β2 integrins, αv-class integrins, and scavenger receptors. Those contacts change spreading, actomyosin tension, metabolism, and phagocytosis, sometimes before a conventional cytokine phenotype is apparent. A single CD86/CD206 value cannot distinguish these functions. It does not tell us whether cells are being recruited, clearing apoptotic cells, supporting angiogenesis, degrading matrix, or entering a foreign-body giant-cell program. Receptor occupancy, secreted factors, metabolic flux, matrix-remodeling activity, and time-resolved single-cell states give a more informative picture (Xie et al., 2020; Wen et al., 2023; Wan et al., 2024; Abedi et al., 2025; Rahaman and Mukherjee, 2025).

The first adsorbed layer contains fibrinogen, FN, vitronectin, albumin, immunoglobulins, and complement proteins in changing proportions. Conversion of fibrinogen to fibrin then creates a provisional matrix used by monocytes, neutrophils, progenitors, and endothelial cells. Complement fragments and immunoglobulins contribute to opsonization and recruitment, while FN and vitronectin supply integrin ligands. The goal at this stage is not zero inflammation. A useful response clears damaged material and then resolves before the same pathways become chronically active.

Whether the early layer functions as a repair matrix or an injury signal depends partly on protein presentation. Exposing normal adhesion domains can stabilize attachment and support reparative secretion. Excessive structural distortion produces a different outcome: unfolded fibrinogen can engage Mac-1, persistent complement deposition can extend C3a/C5a signaling, and strongly distorted albumin can become a scavenger-receptor ligand (Corbo et al., 2016a; Deng et al., 2011). A screen that measures only MSC adhesion would miss all three risks. Coagulation, platelet activity, complement, neutrophils, and macrophages therefore need to be assessed alongside osteogenic cells.

### 4.2 Angiogenesis and matrix assembly: coupling adhesion to growth-factor signaling

Keeping VEGF at the interface is not the same as building a functional vascular network. The angiogenic sequence includes tip-cell selection and stalk-cell proliferation, then lumen formation, basement-membrane deposition, and pericyte recruitment; pushing one step too strongly can disrupt the next. Exposed FN and VEGF-binding regions may speed early sprouting, but prolonged signaling can leave vessels permeable and poorly matured. PDGF-BB-dependent pericyte recruitment and matrix stabilization then become necessary. Bone imposes an additional condition: more capillaries do not automatically create an osteogenic vascular niche. Perfusion, permeability, vessel maturity, pericyte coverage, and distance from osteoprogenitors are more informative endpoints than tube length alone (Xie et al., 2020; Wen et al., 2023; Wan et al., 2024; Abedi et al., 2025).

In this period, the same ECM is read by two receptor systems with different jobs. Integrins report whether a cell can gain mechanical purchase; growth-factor receptors report whether a morphogen is available locally. When a material drives FN fibrillogenesis, RGD/PHSRN and growth-factor-binding regions can be presented within the same matrix. In selected models, that arrangement improves BMP or VEGF signaling at lower doses than soluble delivery (Llopis-Hernández et al., 2016; Martino et al., 2014; Xiao et al., 2022; Damiati et al., 2022). Calling the result ‘factor retention’ is therefore incomplete. Domain exposure, vessel maturation, and the surrounding protein repertoire all influence whether retained factor is actually functional.

Immune, vascular, and osteogenic cells can reinforce one another during this interval, but the direction of that feedback is not automatically favorable. Macrophage-derived VEGF, PDGF, and matrix-remodeling factors can support sprouting. Endothelial and perivascular cells help form an osteogenic niche, and perfused vessels bring additional plasma proteins and progenitors into the defect. Vessel number by itself is a weak endpoint. What matters is whether the network is perfused, stabilized by adequate pericyte coverage, and positioned to support osteogenesis.

### 4.3 Mineralization and remodeling: control shifts to cell-derived matrix

Mineralization feeds back on the same interface that helped initiate it. Newly deposited calcium phosphate changes local charge, ion activity, roughness, and protein affinity. Fetuin-A, osteopontin, matrix Gla protein, albumin, and other phosphoproteins can then restrain, localize, or template mineral growth. Their effect is spatially conditional: a state that is useful at the bone-contact front may be undesirable in neighboring soft tissue. Normal late repair also requires osteoclast activity. αvβ3-mediated adhesion to RGD-containing bone matrix and formation of sealing zones are part of turnover, not a failure of regeneration. A material that stimulates osteoblasts but prevents remodeling has therefore not completed the repair process (Xie et al., 2020; Wen et al., 2023; Wan et al., 2024; Abedi et al., 2025).

The transition to cell-derived matrix is also altered by disease. Diabetes introduces glycated and oxidized proteins whose receptor affinity and resistance to proteolysis differ from the unmodified forms. Osteoporosis changes turnover, endocrine cues, and circulating mineralization regulators. Aging combines low-grade inflammation with weaker macrophage resolution, poorer angiogenic competence, and shifts in albumin and immunoglobulin profiles. Infection changes the interface again by adding bacterial proteins, toxins, and biofilm matrix that compete with host proteins. These variables are large enough to change the acquired biological identity. A material should either remain effective across the disease population for which it is intended or be paired with an explicit patient-selection strategy.

As mineralization proceeds, cells add type I collagen, osteopontin, osteocalcin, and other mineral-binding proteins to the surface. This cell-derived matrix gradually displaces the importance of the initial corona. The material now has to permit mature focal adhesions, traction, collagen organization, and apatite nucleation while avoiding persistent stimulation, ectopic ossification, and chronic inflammation.

Late repair also requires release from some of the strong interactions that were useful earlier. Amplified signaling can help during the first phase, but later control should shift toward osteoblast-osteoclast coupling and mechanical adaptation. Permanently fixing growth factors at high affinity can obstruct turnover; allowing them to exchange too quickly removes spatial control. Reversible anchoring or phase-dependent degradation may fit this change better, but direct evidence that endogenous protein exchange can be deliberately programmed in this way is still limited.

## 5 Core protein modules and their multicellular effects

The next sections use specific protein modules to test the proposed causal chain rather than simply listing adsorbed proteins. For each module, we ask what protein state was actually measured and whether a receptor-level perturbation tested causality. We also record the experimental domain in which the effect was shown. The remaining question is then where the argument still crosses an evidence boundary: from purified protein to authentic biofluid, from nanoparticles to bulk implants, or from animal data to human bone healing.

### 5.1 Fibronectin and vitronectin: integrin selectivity as a cell-fate regulator

FN offers the clearest protein-state example because its integrin-and growth-factor-binding regions can be studied separately. The RGD motif in FNIII10 and the PHSRN synergy site in FNIII9 favor α5β1 recognition when their spatial relationship is preserved. Defined surface-chemistry studies show that adsorption can change FN presentation, integrin preference, and focal-adhesion signaling even when total attachment is similar. Titanium studies also associate FN organization with osteoblastic responses (Keselowsky et al., 2003; Michael et al., 2003; Keselowsky et al., 2004; Keselowsky et al., 2007b; Moursi et al., 1997; Salasznyk et al., 2004; Martino et al., 2009). Material-driven FN/vitronectin assembly can improve BMP-2 presentation as well (Xiao et al., 2022). The limitation is scale: most of these causal chains are strongest in IV systems and do not yet show that FN presentation is necessary for load-bearing bone healing.

Vitronectin is abundant in plasma and participates in adhesion, complement regulation, coagulation, and matrix organization. It binds αv integrins and can compete or cooperate with FN. The biological outcome depends on relative abundance, assembly, and cell type. Endothelial cells, macrophages, osteoblast-lineage cells, and osteoclasts express different integrin repertoires that change with maturation. A surface cannot be described as broadly ‘cell adhesive’ without specifying which receptor is engaged in which cell. Competitive blocking, receptor knockdown, and integrin-specific tension sensors are more informative than total attachment. (Keselowsky et al., 2003; Deng et al., 2011; Llopis-Hernández et al., 2016).

At a mineralizing surface, osteopontin and other bone-matrix proteins become more prominent. Osteopontin contains an RGD motif, and proteolysis can expose additional cryptic sequences that connect mineral surfaces with integrins and CD44. Its localization at osteoclast-bone interfaces is consistent with a role in anchorage and remodeling (Reinholt et al., 1990). Yet the same protein also restrains uncontrolled crystal growth. It can therefore support cell attachment and osteoclast function while limiting mineral expansion. For this reason, abundance alone is not enough; proteolytic state and domain accessibility need to be measured as well.

### 5.2 Coagulation, complement, and immunoglobulins: repair signals with safety limits

Fibrinogen is a useful stress test for any claim about benign protein restructuring because it responds strongly to hydrophobicity, charge, and nanoscale curvature. Classic biomaterial studies connected fibrin (ogen) with acute inflammatory recruitment and identified a cryptic fibrinogen sequence that supports Mac-1-dependent leukocyte adhesion (Tang and Eaton, 1993; Tang et al., 1996; Hu et al., 2001). When adsorption unfolds the protein, the γ-chain region recognized by Mac-1 can become exposed, increasing inflammatory-cell adhesion and cytokine release (Deng et al., 2011). Thrombin cleavage then creates a different structure altogether: fibrin forms a network whose fiber diameter, branching, stiffness, and susceptibility to plasmin depend on local chemistry and protein concentration. That network controls migration and growth-factor retention. A measurement taken only at the fibrinogen adsorption step therefore misses later parts of the mechanism. The biologically relevant sequence runs from adsorption and structural change through enzymatic conversion, network formation, receptor binding, and degradation.

Complement adds several distinct readouts to the early interface. Activation can proceed through classical, lectin, or alternative routes, and a material can shift those routes by recruiting immunoglobulins, C3 fragments, regulators, or pattern-recognition molecules. Studies linking biomaterial-associated complement composition with macrophage behavior do not give one consistent direction; the result changes with the fragment measured, material, donor fluid, and assay conditions (Araújo-Gomes et al., 2019). Limited activation may assist recruitment. Sustained activation, by contrast, generates anaphylatoxins and membrane-attack complexes and can produce vascular effects or rapid clearance. Total C3 adsorption cannot tell whether the surface carries intact C3, C3b/iC3b, or active factor H. Functional interpretation therefore needs measurements such as C3a, C5a, SC5b-9, factor-H recruitment, hemolytic activity, and receptor-dependent cellular responses.

For immunoglobulins, the orientation of the adsorbed molecule changes what the immune system can read. An Fc region exposed to the fluid can engage Fc receptors and promote opsonization of a particle or implant. If Fc faces the material, Fab regions may remain accessible while phagocytic recognition falls. The host state adds further variation: autoantibodies, monoclonal therapies, and altered glycosylation can all modify Fc-receptor signaling. Medication therefore belongs in the description of the biointerface, not only in the demographic table.

Coagulation, complement, and immunoglobulin signaling each have a useful operating range. Suppressing them completely would also remove functions needed for recruitment and debris clearance. The opposite extreme is equally problematic: persistent activity increases thrombosis, inflammatory amplification, and fibrosis. Interface design should therefore be judged by whether these pathways resolve at the appropriate time as well as by whether they are activated initially.

### 5.3 Albumin and transport proteins: passive shield or altered ligand?

Albumin is often used to block free surface area, but its behavior in patient biofluid is more complicated than a passive coating. It may be displaced by later proteins, remain beneath an outer layer, or unfold enough to acquire new receptor interactions. Its fatty-acid-and drug-binding sites introduce another source of variation because medication, lipid status, and oxidation can change adsorption behavior. A pre-adsorbed albumin film in buffer is therefore a poor stand-in for albumin recruited from plasma. The useful measurements are functional ones: whether native ligand-binding and redox activities are retained, and whether anchored albumin suppresses or redirects subsequent protein exchange (Keselowsky et al., 2003; Deng et al., 2011; Llopis-Hernández et al., 2016).

Fetuin-A is particularly relevant on calcium-phosphate and other mineral-based materials because it binds calcium-phosphate nanoclusters and contributes to calciprotein-particle formation. In doing so, it helps prevent indiscriminate mineral precipitation (Heiss et al., 2003). On hydroxyapatite or magnetic scaffolds, enrichment of fetuin-A may therefore change mineralization and immune recognition at the same time (Zhu et al., 2017; Zhu et al., 2019). Systemic inflammation or renal disease can lower fetuin-A, which means the acquired layer may change in both amount and character. Transferrin, apolipoproteins, and complement regulators make a related point: a component that is not abundant can still dominate uptake or immune fate if it engages a high-value receptor.

### 5.4 Growth-factor-binding ECM and bone-matrix proteins: presentation at lower dose

Growth-factor presentation is better separated into four processes: capture, protection from proteolysis, control of diffusion, and display in a form the receptor can reach. Improving one can worsen another. Very strong binding retains a ligand but may prevent release or hide its receptor-binding surface; weak binding preserves mobility but may fail to localize the signal. Fibrin (ogen) heparin-binding regions and related ECM motifs provide examples of intermediate-affinity reservoirs that can support repair (Lee et al., 2011; Martino et al., 2013). Engineered surfaces can still impose a density or orientation that does not occur physiologically. Surface-presented and soluble factor should therefore be compared over dose-response curves and under receptor-blocking conditions (Llopis-Hernández et al., 2016).

BMP-2 shows why lower-dose presentation is a translational issue rather than simply an efficiency claim. Supraphysiological rhBMP-2 can generate substantial bone, but inflammation, ectopic bone, osteolysis, edema, and other context-dependent complications have also been reported (James et al., 2016). Experimental FN nanonetworks and ECM-affinity strategies can increase local BMP activity while reducing the required dose (Llopis-Hernández et al., 2016; Xiao et al., 2022; Martino et al., 2014). That evidence does not automatically establish endogenous recruitment. Adult wound fluid contains much less BMP than common experimental loading solutions and also contains antagonists. A material described as ‘growth-factor-free’ should therefore show how much active ligand it captures, whether the relevant receptor is phosphorylated, and whether loss of the recruited ligand removes the response.

In one FN-assisted presentation study, polymer-coated sleeves carried approximately 15 ng of recombinant BMP-2 at a measured surface density of 100 ± 8 ng/cm^2^ and were tested in a 2.5-mm mouse radial defect (Llopis-Hernández et al., 2016). The study also used a 25 ng/mL BMP-2 adsorption solution in its *in vitro* protocol. Solution concentration, retained surface density, and total implant dose are distinct quantities; none measures endogenous BMP-2 capture. The recombinant-factor system provides a mechanistic and bone-relevant experimental example, not a clinical dose-equivalence rule.

VEGF has a related timing problem. Capturing it early may help vascularize a large defect, but maintaining a high local level for too long increases permeability and can leave vessels immature. One plausible sequence would be early VEGF capture, subsequent PDGF-associated stabilization, and then a decline in angiogenic signaling. Endogenous protein exchange could, in principle, generate that sequence without several exogenous payloads. Direct evidence is still sparse, so we regard this as a design hypothesis rather than an established mechanism.

Endogenous recruitment must be distinguished from loading a low dose of recombinant factor. A genuinely endogenous mechanism requires quantification of active ligand captured from patient-derived fluid or the local wound milieu and loss-of-function evidence showing that the recruited ligand is necessary for the response.

Testing endogenous capture requires a mass balance using the starting fluid volume, unbound fraction, wash losses, and retained ligand, with process blanks, matrix-matched calibration, spike recovery, and limits of detection or quantification. Immunoreactive mass should then be linked to activity using a matched soluble BMP-2 dose-response in the same cells and observation window, together with BMP-2 neutralization or receptor blockade and an early readout such as SMAD1/5 phosphorylation. Such a comparison may establish an assay-specific activity equivalent; it does not establish equivalence to a delivered clinical dose. A validated amount and activity of endogenous BMP-2 sufficient for bone healing across implants cannot be inferred from the recombinant loading examples.

The beneficial states, principal risks, and validation requirements of major protein modules are compared in Table 4.

**TABLE 4:** Protein modules, potential functions, risks, and validation requirements.

Protein module	Potentially beneficial state	Major risk	Critical validation
FN/vitronectin	Accessible FN RGD/PHSRN regions; vitronectin RGD and appropriate receptor presentation	Epitope shielding; excessive adhesion; bacterial use of host ligands	Protein-specific accessibility; α5β1/αv blockade; host-cell and bacterial assays
Fibrinogen/fibrin	Provisional matrix for hemostasis and cell recruitment	Unfolding, Mac-1 activation, thrombosis, chronic inflammation; bacterial attachment	Conformation-sensitive antibody; coagulation; platelet and Mac-1 blockade
Complement/Ig	Opsonization, recruitment, and debris clearance	C3a/C5a excess, phagocytic clearance, inflammatory amplification	C3 fragment deposition, SC5b-9, complement inhibition
Albumin/transport proteins	Passivation and controlled transport of lipids or ions	Denaturation and scavenger-receptor recognition	Secondary structure, aggregation, SR-A/MARCO dependency
Growth-factor-binding ECM	Localized storage and receptor-cooperative presentation	Ectopic bone, abnormal vessels, sustained signaling	Spatial receptor activation, exchange kinetics, low-dose *in vivo* efficacy
Autologous PRP/PRF	Patient-derived provisional matrix and preparation-dependent local factor release	Donor/protocol variation; rapid loss; unproven indication-specific benefit	Composition, fibrin structure, retained dose, release, and controlled clinical outcomes

Potential benefit is conditional on the experimental and clinical setting. PRP/PRF, applied before implantation is an autologous precoat; it is distinct from selective host-fluid recruitment after implantation.

## 6 Material properties that alter protein adsorption and functional presentation

Material design is easier to interpret when the intended interface function is separated from the material feature used to create it. Higher surface free energy is a feature. Exposing the FN synergy site while avoiding inflammatory fibrinogen unfolding is a function. A nanoscale ridge is also a feature; receptor-ligand spacing that supports integrin clustering is the biological output of interest. Keeping these levels separate reduces *post hoc* explanations in which a favorable response is assigned to whichever material property appears most conspicuous. A stronger experiment begins with a target interfacial protein state, predicts how a material interaction could create it, and then tests whether that protein state mediates the response (Barberi and Spriano, 2021; Lin et al., 2022; Zhu et al., 2019).

Material variables rarely change one at a time. UV treatment can change carbon contamination, hydroxyl density, wettability, and surface energy in the same experiment. Adding ions to a ceramic can alter charge, dissolution, pH, and protein affinity together. Nanotopography changes accessible area, curvature, and local mechanics at once. A single-factor explanation is therefore weak unless matched controls separate these co-varying properties. Factorial designs are one option. Another is an orthogonal comparison: use chemically different materials that generate a similar interfacial protein state, or use closely matched surfaces that differ in interfacial protein state while roughness and mechanics remain similar. Agreement across both tests gives stronger support for a protein-mediated mechanism.

The relative abundance of a target protein may increase either through increased target adsorption or through reduced recovery of other proteins. Absolute surface density is therefore needed to distinguish greater capture from a change in the denominator. Studies should report absolute surface density as well as relative composition, with controls for recovery and accessible area. A favorable target/off-target ratio also does not guarantee safety. A small amount of a potent procoagulant or complement-activating species can dominate the functional response. Optimization should be based on functional thresholds rather than rank order alone (Barberi and Spriano, 2021; Lin et al., 2022; Zhu et al., 2019).

No single surface chemistry is likely to hold every protein in a desirable state. A hydrophobic interaction that stabilizes one assembly may denature another protein. Heavy hydration can suppress nonspecific adsorption but also exclude ECM proteins required for adhesion. Covalent capture improves retention but may prevent normal exchange and remodeling. The design problem therefore has several objectives at once. Osteogenic adhesion, immune resolution, vascular maturation, coagulation and complement safety, bacterial competition, and manufacturability all need to be considered. A surface that balances these outcomes may have more clinical value than one that maximizes a single *in vitro* endpoint (Barberi and Spriano, 2021; Lin et al., 2022; Zhu et al., 2019).

### 6.1 Surface energy, wettability, and chemical groups

Hydrophilicity often accompanies better early osseointegration, but wettability itself does not identify the causal route. Titanium and titanium-associated coatings show why. Surface aging, chemistry, roughness, and post-processing can each change adsorption, FN organization, integrin engagement, osteoblast attachment, and *in vivo* osseointegration (Bumgardner et al., 2003; Lüthen et al., 2005; Att et al., 2009; Ueno et al., 2012). After UV treatment, higher surface free energy and hydroxyl density can occur together with changes in FN amount and secondary structure, α5/β1 integrin expression, Rac1 signaling, alkaline phosphatase activity, and mineralization (Yang et al., 2003; Lin et al., 2022). Those correlations do not reveal which change is carrying the effect. Protein depletion, conformational controls, integrin blockade, and matched controls for roughness, contamination, ion release, and sterilization are needed to separate the routes.

### 6.2 Curvature and nanoscale topology

Curvature changes both how much of a protein can contact the surface and how much deformation is energetically favorable. A highly curved small nanoparticle may restrict the contact area of one large protein while increasing crowding between neighboring proteins. A flatter surface can allow the same molecule to spread more extensively. Nanowires, nanopores, and hierarchical roughness add local electric fields, capillary effects, and confinement, all of which can change exchange kinetics. For this reason, adsorption should be related to true accessible area rather than only to projected geometric area.

### 6.3 Charge, ion coordination, and mineral binding

A single net-charge value misses the patchy electrostatics of both proteins and material surfaces. Even an overall negatively charged protein can bind a negatively charged surface if local positive domains, counterion release, hydrophobic contacts, or cation bridges provide enough favorable interaction. Calcium and other multivalent ions can also reorganize hydration and bridge carboxylate or phosphate groups. Zeta potential therefore has limited value as a stand-alone predictor. Molecular simulation, site-directed protein variants, and ion-substitution experiments can test which domain or coordination interaction is actually responsible for binding (Barberi and Spriano, 2021; Lin et al., 2022; Zhu et al., 2019).

Dissolution changes more than which proteins are recruited; it can chemically modify the adsorbed proteins themselves. Reactive oxygen species generated by some nanomaterials can oxidize methionine, cysteine, or aromatic residues. Metal ions may catalyze oxidation or cross-linking, whereas local alkalinization around bioactive glass changes protein charge and protease activity. What appears to be a purely ‘conformational’ effect may therefore include covalent modification. When these reactions are plausible, proteomic workflows should search for oxidation, glycation, deamidation, and cleavage rather than assuming that the adsorbed sequence remains chemically native.

Ca2+, Mg2+, Zn2+, and phosphate groups interact with carboxylates, phosphorylated residues, and metal-binding domains. Hydroxyapatite has intrinsic affinity for many bone-associated proteins. Magnetic hydroxyapatite has been reported to generate a distinct corona and to enhance proliferation through MAPK signaling (Zhu et al., 2017; Zhu et al., 2019). However, ion release, magnetic effects, and corona-mediated effects can coexist. Corona depletion or replacement, ion-matched controls, and pathway blockade are required to partition their contributions.

### 6.4 Polymer chain chemistry, dynamic bonds, and ECM-like assembly

PEA provides a useful counterexample to the assumption that chemically similar polymers produce similar protein states. On PEA, FN can move from a compact adsorbed form into nanofibrillar networks, while related polymers do not always generate the same assembly (Llopis-Hernández et al., 2016; Xiao et al., 2022; Damiati et al., 2022). Dynamic boronate ester, imine, host-guest, catechol, and metal-coordinate chemistries offer another route to reversible anchoring in response to pH, reactive oxygen species, enzymes, or load. The risk is that the same chemistry can modify lysine, cysteine, or other residues needed for protein function. Protein integrity therefore needs to be measured directly rather than inferred from material responsiveness.

Protein integrity should be assessed at the chemical, structural, and functional levels. Reducing and nonreducing electrophoresis or immunoblotting can screen for cleavage and covalent aggregation, while intact-mass analysis or LC-MS/MS peptide mapping can identify chemical modifications. Where feasible, these measurements should be paired with *in situ* spectroscopy and site-accessibility assays. Retained activity requires a protein-specific test, such as integrin binding and blockade for FN or BMP-2-dependent SMAD1/5 phosphorylation with a neutralization control. Comparisons should account for the amount of protein retained and include native, deliberately damaged, and recovery-matched controls. Assays requiring desorption should be interpreted alongside measurements made on the intact interface. These are proposed validation requirements, with complementary proteomic and structural methods discussed in Sections 8.1 and 8.2.

### 6.5 Spatial patterning across a heterogeneous defect

A bone defect does not expose every part of a scaffold to the same biological problem. Perivascular regions need endothelial adhesion and vessel stabilization. A mineralizing front needs osteogenic integrins and controlled apatite nucleation. Areas at high risk of infection must also resist bacterial colonization. Charge gradients, patterned nanotopography, or region-specific ligands could therefore be used to create different local interfacial protein states on one scaffold. Under this view, precision means matching an interface state to its tissue compartment, not identifying a single corona for the entire defect.

## 7 From protein state to cellular networks: receptors and signaling hubs

For interpretation, it helps to separate receptor-to-tissue responses into four levels without assuming that they always occur in a straight line. At the first level, exposed protein domains bind integrins, complement and Fc receptors, scavenger receptors, or growth-factor receptors. At the second, ligand spacing and mobility shape receptor clustering, co-localization, and endocytosis. Intracellular pathways form a third level, where FAK/Src, Rho GTPases, PI3K/Akt, MAPK, NF-κB, Smads, and calcium signaling can converge. The fourth level is intercellular spread through cytokines, extracellular vesicles, ECM deposition, proteases, and vascular transport. One material can act at several of these levels. A causal claim is easier to defend when the experiment identifies the earliest level at which a reproducible change can be demonstrated (Keselowsky et al., 2004; Martino et al., 2009; Deng et al., 2011).

Integrin signaling is constrained by mechanics. Ligands that remain stably anchored allow cells to generate traction, recruit talin and vinculin, mature focal adhesions, and activate FAK/Src and RhoA. This provides a route from domain accessibility to context-dependent YAP/TAZ-RUNX2 activity. The same mechanism can become maladaptive if adhesion or tension remains excessive and favors fibrosis or limits migration. Endpoint attachment cannot distinguish these possibilities. Receptor occupancy, traction or tension sensors, and time-resolved signaling are more informative (Keselowsky et al., 2004; Martino et al., 2009; Deng et al., 2011).

Several downstream pathways can carry the signal, but they do not identify the initiating interface event by themselves. FAK/Src and PI3K/Akt contribute to survival and cytoskeletal responses, whereas ERK, p38, JNK, Smads, and calcium pathways integrate growth-factor and stress inputs. Integrin and growth-factor signaling also interact rather than running as separate channels. FN nanonetworks can position BMP-2 or VEGF near mechanically competent adhesion sites and thereby increase cooperation among Smad, ERK, and PI3K/Akt signaling (Llopis-Hernández et al., 2016; Xiao et al., 2022; Martino et al., 2014). A later increase in RUNX2 or alkaline phosphatase is compatible with this route, but it is not proof of it. Earlier receptor phosphorylation, spatial co-localization, and functional inhibition test the mechanism more directly.

Immune signaling at the interface cannot be summarized by a single polarization label. Structurally changed fibrinogen may engage Mac-1, an appropriately oriented immunoglobulin can bind Fc receptors, and iC3b can signal through complement receptors, with effects on phagocytosis and inflammatory signaling. Some biomaterials also activate inflammasome-or CSF-1R-dependent programs that do not require an adsorption-based explanation (Malik et al., 2011; Doloff et al., 2017; Keselowsky et al., 2007a). An M1/M2 readout therefore leaves out much of the response. Receptor dependence, secretome, metabolism, efferocytosis, matrix remodeling, and foreign-body-associated states should be followed over time. When NF-κB or macrophage responses are interpreted, endotoxin controls remain essential.

Cell state changes with time, so a late phenotype cannot automatically be used to infer the initiating mechanism. Early inflammatory signaling helps recruit cells and can contribute to angiogenesis. Intermediate states support efferocytosis and matrix formation. If the same network stays active, however, it can later promote fibrosis, osteoclastogenesis, or implant loosening. Time-resolved single-cell data combined with blockade or rescue can help separate an initiating event from a late correlate. Vascular measurements need the same caution. CD31 area alone does not establish vessel maturity or function; Emcn, pericyte coverage, perfusion, permeability, and oxygenation give a more direct description of vascular quality.

## 8 Methods for testing an anchoring-conformation-function causal chain

Interface measurements can change the interface they are meant to describe. *Ex situ* recovery gives molecular detail but often removes weak interactions. *In situ* methods preserve the local setting better, but usually provide less molecular specificity. No single technique solves both problems. Stronger studies therefore combine methods that address different steps of the causal chain: adsorption and displacement in real time on model surfaces, low-perturbation recovery from complex fluid, proteomic and structural mapping, receptor-level perturbation, and spatial analysis *in vivo*. A new technology adds value only when it closes a defined evidentiary gap rather than adding another descriptive layer (Pisani et al., 2017; Weber et al., 2019; Bashiri et al., 2023; Mahmoudi et al., 2013).

### 8.1 Quantitative proteomics and proteoform-level analysis

Bottom-up proteomics gives broad coverage by identifying peptides after enzymatic digestion. The cost is loss of some information about the intact proteoform and about whether a peptide came from a cleaved fragment. Data-independent acquisition improves reproducibility across samples, and isotopic or isobaric labels allow multiplexed comparisons. Top-down and middle-down approaches retain more information about intact mass and combinations of modifications, but throughput and sensitivity are still limiting. Once candidate proteins have been identified, targeted parallel-reaction monitoring is useful for testing them across larger donor cohorts.

Statistical enrichment and biological sufficiency should not be treated as the same result. A BMP-related peptide may be significantly enriched and still remain below the concentration needed to activate its receptor. Conversely, a modest shift in a complement regulator can have a large functional effect.

Quantitative proteomics is easier to interpret when abundance is normalized to accessible area and, where feasible, reported as molecules per particle or per unit scaffold area. Sample preparation adds another source of variation. If corona proteomics is ever used as a product-release assay, shared reference materials and interlaboratory ring trials will be needed to show that the measurement transfers between laboratories.

Different proteomic formats preserve different information. DIA and label-based LC-MS/MS quantify composition, whereas top-down approaches retain more detail about cleavage, glycosylation, oxidation, and other proteoforms. Yet the apparent corona can change substantially with the recovery workflow itself. Washing strength, centrifugation or magnetic isolation, digestion efficiency, and database strategy all affect what is recovered (Bai et al., 2021; Pisani et al., 2017; Weber et al., 2019; Bashiri et al., 2023). Process blanks, biofluid controls, recovery standards, and inter-batch quality controls are therefore part of the measurement, not optional extras. A protein list without quantitative recovery information is difficult to compare with one generated by another protocol.

### 8.2 Measuring conformation and orientation

Hydrogen-deuterium exchange mass spectrometry can localize changes in solvent accessibility and backbone dynamics, while limited proteolysis shows which regions become more exposed or protected. Cross-linking mass spectrometry adds distance constraints and can identify protein-protein assemblies. Applying these methods to insoluble materials is difficult because the desorption step itself may change protein structure. One control is to desorb all comparison groups in the same way and include both native and deliberately denatured references. Without that control, a structural change produced during retrieval can be mistaken for a change caused by adsorption.

Conformation-sensitive monoclonal antibodies can provide high-throughput site-specific readouts, but an antibody epitope is not identical to a receptor-binding surface and antibody access can be influenced by steric crowding. Single-molecule force spectroscopy measures how strongly a receptor or antibody binds to the anchored protein and can distinguish heterogeneous populations. Super-resolution microscopy and DNA-PAINT-like approaches may eventually map domain accessibility and ligand spacing on clinically relevant surfaces, although labeling can perturb the interface.

For an FN example, HFN7.1 binding can be reported after background subtraction and normalization to the adsorbed FN amount, with a same-batch reference surface and the antibody concentration and detection range specified (Llopis-Hernández et al., 2013). The result is a relative accessibility readout, not an absolute percentage of exposed integrin-binding sites without calibration. Orientation, conformation, and steric crowding can all affect it. Pairing the readout with structural imaging and integrin binding or blockade therefore tests its functional interpretation. Protein-integrity assessment should also distinguish chemical damage from altered presentation, using the complementary controls described in Section 6.4.

Structural techniques do not answer the same question. ATR-FTIR and circular dichroism give ensemble information about secondary structure. Conformation-sensitive antibodies examine selected epitopes. Hydrogen-deuterium exchange, limited proteolysis, and cross-linking mass spectrometry add regional accessibility or distance constraints. Atomic-force and single-molecule force measurements probe adhesion or unfolding forces, while ToF-SIMS, surface-enhanced spectroscopy, and oriented isotope labeling can inform molecular orientation. None of these measurements demonstrates receptor function on its own. The most useful interpretation combines a global structural readout, a site-accessibility measurement, and a receptor-dependent functional test.

### 8.3 Following the interface over time and *in situ*

A static incubation omits both flow and repeated delivery of fresh proteins. Microfluidic systems can introduce shear, expose a surface to fluids in sequence—for example, blood followed by a marrow-like medium—and add endothelial barriers or immune cells. QCM-D measures coupled mass together with viscoelastic behavior, which can help distinguish a compact layer from a highly hydrated one. The fitted model, however, is not a unique structural solution. SPR adds kinetic information, but refractive-index changes and mass transport can also influence the signal (Pisani et al., 2017; Weber et al., 2019; Bashiri et al., 2023; Mahmoudi et al., 2013).

An interface recovered after *in vivo* exposure is biologically more realistic than one formed only in buffer, but the recovery procedure can introduce new artifacts. Magnetic nanoparticles or removable implant coupons allow sampling at defined times. Surgery, washing, and tissue homogenization can contaminate the recovered material or remove weakly associated proteins. Spatial approaches reduce some of that pooling. Multiplexed imaging can localize proteins and cells, imaging mass spectrometry can map molecular distributions, and proximity labeling may capture species close to an engineered surface. Each approach still needs a control for proteins released when cells are injured during retrieval.

Kinetic tools such as QCM-D, SPR, and ellipsometry are useful for following adsorption and displacement, although most experiments use planar substrates and simplified fluids. Microfluidics can add shear, concentration gradients, and proteins secreted by cells. Organ-on-chip systems go further by combining vascular perfusion, immune and osteogenic cells, and mechanical loading. Recoverable implants or magnetic particles offer another route to measuring an authentic *in vivo* corona (Zhu et al., 2017; Zhu et al., 2019; Bai et al., 2021). A remaining technical gap is the ability to preserve weak soft-corona species and short-lived conformations during *in situ* measurement.

### 8.4 Multi-omics and computational models

Around an implant, single-cell RNA sequencing can resolve changes in immune, endothelial, stromal, and osteogenic populations. Spatial transcriptomics adds location, and single-cell ATAC-seq can test whether early signaling is followed by persistent shifts in chromatin accessibility. These data are more useful when they are linked to proteomics from the same interface. Ligand-receptor algorithms can propose a connection between the transcriptomic and proteomic layers, but an RNA signal does not show that the ligand was secreted or that the receptor was active. A functional perturbation is still needed to test that step.

Microphysiological models are useful because they allow a specific mechanism to be perturbed without removing multicellular context. A vascularized bone-on-chip, for example, can combine perfusion, macrophages, MSCs, osteoblasts, osteoclast precursors, and a replaceable material insert. Biosensors can follow cytokines, pH, oxygen, and physical changes while the experiment is running. Kinase inhibitors, CRISPR perturbations, receptor-blocking antibodies, or RNA interference can then be applied to selected network nodes. The relevant advantage is not simply that the model contains many components; it is that those components remain present while necessity or rescue is tested.

For this problem, machine learning should be framed as a prediction exercise that will be tested prospectively, not as a way to classify a small retrospective dataset. Candidate inputs include surface chemistry, curvature, donor-fluid composition, adsorption kinetics, proteomic composition, and structural features. The output could be receptor engagement or a later bone-healing endpoint. External validation has to prevent information leakage. One option is to withhold an entire chemical family; donor identity should also be modeled hierarchically when repeated samples are used. Otherwise, material similarity or repeated measurements from the same donor can make performance look stronger than it is. If externally validated, an interpretable model might help reduce the interface to a small set of protein-state features suitable for CQA testing.

Molecular dynamics can examine how curvature, charge, and functional groups change adsorption energy or domain exposure, but the result is constrained by the force field, accessible timescale, and the difficulty of representing a complex biofluid. Machine learning faces a different limitation: linking material descriptors, proteomics, conformational measurements, and cell outcomes is useful only when the input data are standardized and tested externally. Spatial transcriptomics, imaging mass spectrometry, and multiplexed microscopy add location to these analyses by mapping cells and proteins around an implant. Recent single-cell and spatial-transcriptomic studies show how these tools can localize osteoimmune and stromal programs around regenerative scaffolds (Wan et al., 2024; Kim et al., 2024).

A causal validation ladder for interface-to-tissue claims is presented in Table 5.

**TABLE 5:** Evidence requirements for a protein-mediated mechanism.

Evidence level	Question	Minimum requirement	Causal strength
I. Interface formation	Is the target protein enriched?	Complex biofluid, multiple times, quantitative proteomics	Association
II. State alteration	Are orientation, conformation, or assembly changed?	At least two complementary structural/epitope methods	Mechanistically relevant
III. Receptor translation	Which receptor reads the state?	Receptor blockade/knockdown, early phosphorylation, co-localization	Moderate-to-strong
IV. Cellular network	Are immune, vascular, and osteogenic functions coupled?	Multicellular models and functional outputs	Systems-level
V. *In vivo* necessity	Is the interfacial protein state required for regeneration?	Protein depletion/replacement, rescue, time-resolved *in vivo* study	Strong
VI. Human translation	Does the mechanism remain relevant in the intended patient population?	Stratified human fluids and prospective clinical-outcome validation on the final-process product	Clinical relevance requires direct outcome evidence

Levels describe complementary evidence requirements; they are not a validated numerical grading scale. Receptor blockade, depletion, and rescue should be interpreted with specificity and material controls.

## 9 Discussion

Figure 3 links the discussion points in Sections 9.1–9.8 to the review’s central claim and the proposed validation sequence in Section 9.9. It shows how causal testing, biological context, safety, and product reproducibility constrain conclusions about protein-mediated bone repair.

**FIGURE 3:**
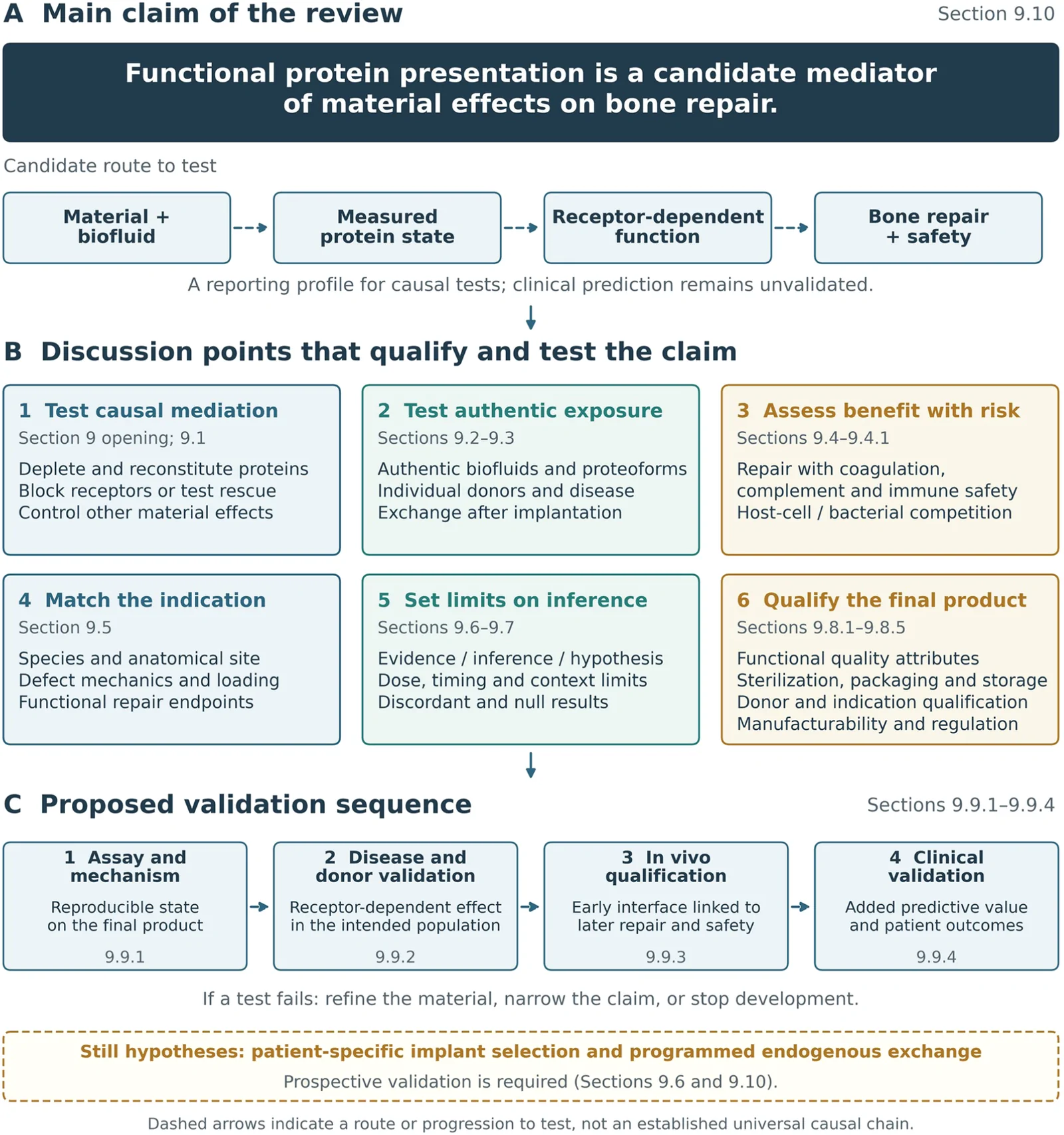
Linking the discussion to the central claim and its validation requirements. **(A)** Functional protein presentation is a candidate mediator of material effects on bone repair. **(B)** The six groups map Section 9 to causal tests, authentic exposure, safety, model relevance, limits of inference, and final-product qualification. **(C)** Sections 9.9.1–9.9.4 propose progression from assay qualification through donor and *in vivo* testing to clinical validation. Failure should prompt refinement, a narrower claim, or discontinuation. Connectors organize the argument; dashed arrows mark routes or progression requiring testing, not a universal causal chain established in one model. Patient-specific selection and programmed endogenous exchange remain hypotheses. The five-dimensional profile is a reporting scheme whose clinical predictive value remains unvalidated.

A central unresolved issue is whether the adsorbed protein layer actually mediates a biological effect or simply changes alongside other consequences of surface chemistry. Both routes can operate in the same material. A surface may alter membranes or release ions while it also changes the protein layer. The two explanations can be separated only by perturbations that uncouple them: change the corona while keeping the underlying surface fixed, or change the surface while recreating a comparable functional corona. Protein depletion and reconstitution, preformed coronas, exchange experiments, receptor blockade, and rescue studies are practical ways to make that comparison. Without such a test, calling an effect ‘corona-mediated’ goes beyond what the data establish (Bashiri et al., 2023; Cisneros et al., 2024; Sanati et al., 2025).

Hard and soft corona are defined partly by the experimental protocol. A protein can move from one category to the other when washing strength or isolation conditions change, even though its biology has not changed. Weak outer proteins may still dominate receptor recognition because they are the first species exposed to cells. Measuring only the hard fraction is analytically convenient, but it can remove exactly that part of the interface. Reports should therefore state what the isolation procedure retained and avoid treating a method-defined fraction as a fixed biological compartment.

Reductionism creates a second problem. FN conformation is experimentally tractable, but a one-protein model leaves out complement, coagulation, lipids, metabolites, and matrix secreted by cells. Untargeted multi-omics can fail in the opposite direction by producing a large network without a mediator that can be tested. We favor moving back and forth between these scales. Candidate states can first be discovered in realistic fluids, rebuilt in a smaller defined system, tested for receptor dependence, and then returned to an *in vivo* model. If the reduced mechanism disappears once biological complexity is restored, it should not be presented as the dominant explanation.

The vocabulary used for the interface also needs scale-specific limits. Protein corona, conditioning film, adsorbed protein layer, interphase, and provisional matrix describe overlapping but nonidentical structures. ‘Corona’ is best established for nanoparticles. A macroscopic implant instead develops a broader conditioning layer that includes proteins together with lipids, ions, platelets, extracellular vesicles, and later cell-deposited matrix. We use interfacial protein state only for the measurable protein component within that larger interphase. The term is meant to keep attention on function, post-translational state, and exchange over time; it does not imply a discrete shell or exclude non-protein biomolecules (Bashiri et al., 2023; Cisneros et al., 2024; Sanati et al., 2025).

Preforming a corona improves experimental control but also changes the biology being modeled. Incubation with autologous plasma, purified FN, or a defined mixture can reduce batch variation and give the material an immediate biological identity. Washing and storage may then lock in a state that would have exchanged rapidly *in vivo*, and implantation exposes the layer to another round of replacement. We therefore treat a precoating as the starting condition, not as a permanent interface. The informative measurements are its displacement rate and whether later *in vivo* exposure remains biased toward the intended function (Bashiri et al., 2023; Cisneros et al., 2024; Sanati et al., 2025).

The literature is much richer in positive than negative interface results. Some materials probably change protein composition without changing bone healing at all. Others may improve early adhesion but have no effect on mechanical union. When such null results are underreported, almost any material feature can be given a favorable protein mechanism after the outcome is known. We think this problem requires better study design rather than stronger wording. Hypotheses should be prospective, primary outcomes should be defined in advance, biological replication should be adequate, and unsuccessful interface engineering should be published. Without those data, it remains difficult to distinguish a measurement that predicts repair from one that is merely easy to detect.

### 9.1 Conformational reprogramming can be overread

A change in average secondary structure does not show that a particular functional domain has become accessible. It also does not establish an osteogenic mechanism. One common shortcut is to report an FTIR shift beside higher alkaline phosphatase or mineralization and connect the two as a causal sequence. The missing steps are precisely the ones that need testing: epitope accessibility, receptor dependence, or rescue. Without at least one of those links, the structural and osteogenic endpoints remain correlated observations.

### 9.2 Purified proteins do not reproduce authentic biofluids

A purified FN solution can help isolate an adsorption mechanism. It is not equivalent to patient biofluid, where thousands of proteins coexist with lipids, metabolites, proteases, and oxidants. Competitors can shield FN from the surface, and post-translational modifications can change its affinity. We therefore increase biological realism step by step: first a single protein, then a defined mixture, followed by plasma or marrow fluid, donor-stratified patient fluid, and finally an interface recovered after *in vivo* exposure.

### 9.3 Patient-specific coronas weaken a one-material-fits-all model

Donor variation should be visible before an average response is called robust. Pooled plasma is convenient for discovery, but it can hide responders and nonresponders and can produce a composite corona that no individual donor actually forms. Validation should keep samples linked to their donor and document age, sex, diagnosis, medication, renal function, inflammatory status, and sample processing. Repeated sampling can help separate a stable donor effect from a temporary change. Studies of liposomes in humans, including material recovered after *in vivo* circulation, have shown coronas that differ from simplified *ex vivo* preparations. Those findings support testing authentic exposure, but they do not yet show that a patient-specific corona predicts the outcome of a bone implant (Hadjidemetriou et al., 2015; Hadjidemetriou et al., 2019; Cisneros et al., 2024).

Total abundance can also hide the proteoform that is actually biologically active. Glycation changes lysine and arginine residues, shifts charge, reduces susceptibility to proteases, and can change receptor recognition. Oxidation can expose hydrophobic regions and promote aggregation. Immunoglobulin glycosylation modifies Fc-receptor signaling. A panel that reports only total FN or albumin therefore misses part of the donor-to-donor variation. Resolving proteoforms is technically harder, but it will probably be necessary if precision-biomaterial claims are meant to extend beyond average healthy-donor behavior.

Host state can change the acquired biological identity. Age, sex, diet, medication, osteoporosis, diabetes, renal dysfunction, infection, and cancer all alter circulating proteins or their post-translational state (Hajipour et al., 2014). A recent hard-tissue-implant study provides a disease-context example: diabetes caused a different corona to form on titanium and zirconia wear particles, and this altered corona was associated with stronger macrophage receptor interactions, JAK1-STAT3 signaling, inflammatory polarization, osteoclast activation, and particle-associated bone destruction (Mao et al., 2026). This is BA evidence in a wear-particle/osteolysis setting. It does not establish that a preoperative patient-specific interfacial protein profile predicts the success of a regenerative implant. Patient-associated differences may help explain failure mechanisms, but their use for clinical stratification still requires prospective validation.

### 9.4 Regenerative and safety endpoints must be optimized together

Better adhesion or higher RUNX2 expression is not enough to establish safety. Thrombosis, complement activation-related pseudoallergy, chronic foreign-body responses, ectopic ossification, abnormal neurovascular ingrowth, and infection can all offset an osteogenic benefit. Surfaces that recruit TGF-β, coagulation factors, or complement are especially dependent on spatial and temporal control because the same pathways can support repair early and fibrosis or systemic toxicity when activation persists.

#### 9.4.1 Host-cell and bacterial competition for the conditioned interface

Bacterial competition places an additional constraint on functional protein presentation. Staphylococcal fibronectin-binding proteins can exploit FN to engage host α5β1 integrins, while fibrinogen-binding adhesins can support attachment to conditioned surfaces (Sinha et al., 2000; McDevitt et al., 1994). The protein features that support mammalian-cell attachment therefore require parallel assessment for bacterial use. Host-cell adhesion alone cannot establish resistance to bacterial attachment or biofilm development.

Damiati et al. (2022) combined FN/BMP-2 presentation with coated titanium nanotopography and examined mesenchymal stromal cells together with *Pseudomonas aeruginosa*. This provides an experimental example of examining host-cell support and biofilm formation in the same system, but it does not establish clinical infection prevention or efficacy against staphylococci. Because topography, coating, and protein presentation were combined, their contributions need to be separated when the mechanism is transferred to another material.

Relevant validation should include protein-conditioned materials, early attachment and viable bacterial counts normalized to area, later biofilm measurements, and host-cell viability and function. Defined mixtures and clinically relevant fluid should be compared, with *Staphylococcus aureus* or *Staphylococcus* epidermidis included when appropriate to the indication. Protein depletion/reconstitution, adhesin-deficient strains or adhesion blockade, and matched topographical controls can test whether an effect depends on a particular host-protein interaction. These are proposed experiments, not evidence that the general five-dimensional profile predicts infection risk.

### 9.5 Species, anatomical site, and mechanics constrain extrapolation

Another boundary comes from species, anatomical site, and mechanics. Rodent plasma differs from human plasma, and a subcutaneous pocket does not reproduce a load-bearing bone defect. Calvaria, long bone, spine, and alveolar bone also differ in vascular supply, marrow composition, and mechanical demand. A preclinical program should therefore begin by naming the intended indication. The biofluid, cell source, animal model, and loading environment can then be selected to match that indication instead of being chosen mainly for experimental convenience.

### 9.6 Separating established findings from inference and hypothesis

Several findings are supported by convergent experimental evidence. Biomaterials rapidly acquire protein layers; surface chemistry, topography, and processing can change protein composition or structure; FN presentation can shift integrin specificity and focal-adhesion signaling; structurally changed fibrinogen can engage Mac-1-dependent inflammatory pathways; and material-driven FN assembly can improve growth-factor presentation and osteogenic responses in defined systems (Tang and Eaton, 1993; Tang et al., 1996; Keselowsky et al., 2003; Michael et al., 2003; Deng et al., 2011; Llopis-Hernández et al., 2016; Xiao et al., 2022). This evidence supports measuring the interfacial protein state as a design variable. It does not define a universal protein state that predicts bone healing across different material classes.

Three broader propositions remain mechanistic inferences: multidimensional interfacial protein states may help explain differences in in vivo bone healing; protein exchange over time might be engineered to coordinate successive repair functions; and a reduced functional CQA panel might represent clinically relevant interface behavior. These ideas still lack cross-material external validation, indication-matched necessity or rescue experiments, and prospective links to mechanical bone union. We therefore treat them as context-dependent inferences rather than general design laws.

The following hypotheses still require prospective testing: closed-loop control of the interfacial protein state, preoperative patient-specific interfacial protein profile testing, patient-specific implant-surface selection, and replacement of multi-factor delivery with programmed endogenous protein exchange. These remain research proposals rather than validated clinical strategies. Demonstrating clinical value would require prospective cohorts or trials showing analytical reproducibility, predictive information beyond conventional clinical variables, and an improvement in safety or bone-healing outcomes.

### 9.7 Discordant results often reveal boundary conditions

Apparent disagreement between studies can reflect a non-linear response rather than a true contradiction. More protein on a surface does not guarantee greater epitope accessibility. A target protein may be enriched but face the wrong direction, unfold too far, or coexist with a small amount of a highly active procoagulant or complement-activating species. FN again illustrates the boundary: moderate extension and ordered assembly may improve integrin and growth-factor presentation, whereas irreversible denaturation or excessive retention removes that advantage. Inflammation follows the same time-dependent pattern. Brief complement and coagulation activity can aid recruitment and clearance; persistent activity can contribute to thrombosis, injury, osteolysis, or fibrosis. The sign of the effect therefore depends on dose, duration, location, and stage of healing.

Experimental context explains another part of the disagreement. Purified systems remove competitors, proteases, lipids, and disease-specific proteoforms, which can make a single-protein mechanism look larger than it is *in vivo*. Plasma, marrow fluid, or patient-derived biofluids may build a different interface.

Nanoparticles add curvature, transport, uptake, and intracellular trafficking that a fixed macroscopic implant does not share. We do not think conflicting studies should be reconciled by choosing the result that best fits a preferred mechanism. A better use of disagreement is to define the boundary condition. That boundary can be tested with indication-matched *in vivo* studies, authentic biofluids, orthogonal material controls, protein depletion or replacement, and receptor blockade or rescue. Confidence increases when the same protein state predicts the same receptor-dependent outcome in materially different systems.

### 9.8 Clinical translation: from material batch to interface-function batch

Translation should begin with the clinical claim, because different products need different kinds of repair. A spinal fusion implant, for example, may need reliable bridging bone under load. An alveolar graft may be judged by preservation of ridge volume while resorbing in a controlled way. A coated fixation device may need faster osseointegration without increasing infection or ectopic bone. These endpoints are not interchangeable, and neither are the protein functions, safety tests, animal models, or follow-up periods needed to support them. A generic rise in osteogenic markers does not establish any of these product-level outcomes (Cisneros et al., 2024; U.S. Food and Drug Administration, 2026; Sanati et al., 2025).

A tiered CQA scheme could reduce the number of measurements as development progresses. Tier 1 would cover conventional material properties and sterility. Tier 2 would add a small mechanistic panel after exposure to a standardized human protein mixture, such as accessible FN synergy sites, fibrinogen inflammatory epitopes, complement activation, and factor-H recruitment. Tier 3 would test whether those readouts remain stable across stratified patient fluids. Comprehensive proteomics is unlikely to be practical for routine lot release. The eventual panel therefore has to shrink to the few measurements that repeatedly predict function.

Large-animal testing should use the final sterilized product with clinically relevant fixation and defect mechanics. Bone volume alone cannot answer whether the repair is functional or safe. A fuller assessment should include bone-to-implant contact and bridging, mechanical strength under torsion or compression, perfused vasculature, immune histology, ectopic mineralization, and systemic complement or coagulation effects. Measuring the early interface proteome in the same species makes it possible to relate that state to later regeneration. This within-model link is more informative than measuring the corona in one system and the bone outcome in another.

Early clinical studies can also test mechanism rather than only efficacy. Perioperative plasma, wound fluid, or drain fluid can be collected alongside a limited interfacial protein state panel and related to fusion or bone-gain outcomes. Such analyses can challenge or support the donor stratification proposed preclinically. They should be prespecified, however, and kept separate from confirmatory endpoints. Otherwise, small exploratory datasets are likely to generate unstable subgroup claims.

#### 9.8.1 Defining critical quality attributes

Conventional CQAs include composition, particle size, porosity, mechanics, residual solvent, and sterility. A protein-anchoring product adds a different class of attributes: target-protein enrichment, accessible functional epitopes, conformational fingerprints, exchange half-life, complement-activation thresholds, and integrin selectivity. These measurements matter for manufacturing only if they can be converted into validated release assays. A mechanistic observation that cannot be reproduced across lots is not yet a product attribute.

#### 9.8.2 Sterilization, packaging, and storage are part of the mechanism

The proposed mechanism can change during sterilization or storage before the device ever reaches a patient. Irradiation, ethylene oxide, moist heat, and plasma treatment can modify surface groups, free energy, and contamination. Hydrophilic titanium can also recover hydrophobicity during storage. Protein recruitment and protein conformation therefore need to be retested on the final sterilized and packaged product rather than only on a freshly prepared research surface. This requirement is consistent with FDA biocompatibility guidance, which evaluates the finished device in the applicable sterilized state (U.S. Food and Drug Administration, 2026).

#### 9.8.3 Stratified human biofluids and companion testing

At present, patient-derived interfacial protein states are candidate mechanistic biomarkers, not validated selection tests. Candidate surfaces can be challenged with donor panels representing the intended indication, including age-and disease-relevant groups such as osteoporosis or diabetes where appropriate. A simplified readout—target-protein ratio, glycation index, functional epitope exposure, or complement activity—should move toward patient selection only after analytical reproducibility and batch stability are shown, and only if it adds prediction beyond conventional clinical variables. Prospective association with bone-healing or implant outcomes is still required. We would reserve terms such as companion diagnostic or patient-specific implant selection for studies showing that acting on the test changes a predefined clinical endpoint.

#### 9.8.4 Indication-driven preclinical development

Alveolar, cranial, long-bone, and spinal defects differ in vascularity, mechanics, and acceptable risk. Early clinical development should begin with an indication that permits local placement, imaging follow-up, and a reasonably controlled tissue environment while addressing a clear unmet need. Infected, osteoporotic, or large load-bearing defects add biological and mechanical variability and are better treated as later tests of robustness after mechanism and manufacturability have been demonstrated.

#### 9.8.5 Regulatory classification and product simplification

Recruiting endogenous proteins instead of carrying recombinant growth factors may reduce dosing and storage problems, but it does not automatically simplify regulation. The developer still has to show that the induced protein state is reproducible and that it does not create abnormal coagulation, complement, or immune activity. From a translation standpoint, chemically defined surfaces that can be sterilized and manufactured at scale have an advantage over systems that depend on several poorly controlled biological components.

The proposed translational development framework is summarized in Table 6.

**TABLE 6:** Proposed translational development sequence.

Stage	Primary task	Proposed progression criterion	Recommended model
Discovery	Identify beneficial proteins and functional states	Enrichment, epitope exposure, receptor dependency	Defined proteins plus healthy human plasma
Mechanism	Test adsorption–presentation–receptor causality	Blockade abolishes effect; rescue restores effect	Multicellular culture and microfluidics
Disease fit	Assess patient-specific coronas	Cross-donor robustness or a defined responder subgroup	Aging, osteoporosis, and diabetes biofluids
*In vivo*	Prove bone formation, mature vessels, and safety	Micro-CT, mechanics, perfusion, coagulation/complement safety	Indication-matched large animal
Scale-up	Lock surface and interfacial protein state CQAs	Batch consistency after sterilization and throughout shelf life	Final packaged product
Clinical	Test feasibility and patient stratification	Safety, fusion/bone gain, revision, and biomarker outcomes	Mechanistic early-phase trial

These are proposed research and product-development checkpoints. Their ability to predict clinical benefit has not been validated.

### 9.9 Future perspectives

We see the next step as a development sequence with explicit stop criteria, not another list of responsive surface chemistries. A candidate interfacial protein state should advance only if four tests are passed: the state is reproduced on the final material, a receptor-level function depends on it, the effect persists in the intended patient context, and the state predicts an outcome relevant to the chosen indication. A positive cell assay is not enough to satisfy these conditions. The stages below are therefore organized around evidence needed to continue development, not around the novelty of the technology.

Extra material complexity should earn its place by improving the target protein state in a measurable and reproducible way. A surface that responds to several stimuli is not necessarily better if sterilization, storage, lot-to-lot variation, coagulation, or complement cannot be controlled. In many cases, the better engineering solution will be the simplest feature that produces the required state reliably.

A broad clinical claim should not be made before disease context has been tested. Individual donor fluids can show whether a surface loses function in aging, osteoporosis, diabetes, renal dysfunction, inflammation, or relevant drug exposure. When several cell types are needed for the proposed mechanism, vascularized or multicellular microphysiological models can add useful context. These models are good for identifying failure-prone conditions. They do not substitute for an indication-matched animal model or for clinical validation.

Standardization will probably depend on a short set of functional measurements that can be transferred between laboratories. Shared reference materials, explicit preanalytical reporting, testing of the final product, and blinded interlaboratory reproducibility directly address that problem. Adding another omics layer does not help if the causal question is still undefined. The difficult choice is which few readouts stay stable across laboratories, donors, and manufacturing lots while retaining a link to function.

#### 9.9.1 Stage 1: establish the assay and qualify the mechanism

Stage 1 is an assay-qualification step. Standardized surfaces and defined protein mixtures provide experimental control, but authentic human biofluids should be included at this stage as well. Candidate states should be characterized in three ways: absolute abundance or surface density, access to the functional epitope, and exchange kinetics. At least two complementary structural or functional methods should confirm the result. The state must reproduce across independent experiments and on the final sterilized product. If sterilization removes it, or if lot-to-lot variation is unpredictable, that state should not be carried forward as the proposed product mechanism.

#### 9.9.2 Stage 2: disease-adapted donor validation

Stage 2 keeps the donor visible in the analysis. The test system should represent the intended patient population rather than pooled healthy plasma alone. Protein depletion or substitution, receptor blockade, and rescue can then ask whether the candidate state is required for the beneficial response. If the effect appears only in an unplanned minority of donors, the claim has to narrow. The data would no longer support a universal-material mechanism, although they could motivate a prespecified responder hypothesis.

#### 9.9.3 Stage 3: indication-matched *in vivo* qualification

Stage 3 tests the final manufactured and sterilized product in an animal model chosen for the intended anatomical site and mechanical demand. In that same model, the early interfacial protein state should be measured prospectively before later outcomes are assessed, including bone-to-implant contact, bridging, perfusion, mechanical strength, and coagulation/complement safety. For a functional CQA, correlation alone is insufficient. Perturbing the proposed protein-receptor step should also change the later outcome.

#### 9.9.4 Stage 4: clinical testing with mechanistic readouts and reporting standards

Stage 4 asks whether a small prespecified interfacial protein state panel adds information in early clinical studies beyond ordinary clinical variables. Analytical stability and incremental predictive value should be tested against endpoints that matter to patients and products, including fusion, bone gain, osseointegration, revision, or safety. Reproducibility also depends on enough methodological detail to rebuild the interface experiment. Reports should state the final sterilization state; the biofluid source, anticoagulant, and protein concentration; flow and contact time; washing or separation procedures; operational definitions of hard and soft corona; quantitative protein data; conformational or epitope methods; receptor perturbation; donor variables; species and anatomical site; and the mechanical environment. Interfacial protein state-guided implant or surface selection should come only after prospective validation, not before it.

### 9.10 Conclusion

Protein adsorption and functional presentation provide specific routes by which material properties can affect responses relevant to bone repair. Current evidence most clearly supports defined links involving FN presentation, integrin selectivity, growth-factor presentation, and fibrinogen-dependent inflammation. These links arise in different experimental settings and do not together establish a universal causal sequence from an endogenous protein layer to successful bone healing. Purified precoatings, exogenous recombinant-factor presentation, dispersed particles, and authentic host-fluid recruitment must retain their separate evidence boundaries.

The five-dimensional profile proposed here is a reporting and comparison scheme, not a validated predictor or a composite clinical score. Its use requires matched exposure conditions, explicit missing measurements, and separate tests of receptor-dependent function. Condition-specific quantitative anchors can guide experimental design, but they are not universal therapeutic targets. Autologous blood-product coatings and bacterial competition further illustrate why protein content or mammalian-cell adhesion alone cannot establish clinical benefit. Future studies should connect measurements on the final material to causal perturbations, disease-relevant biofluids, safety, and indication-matched outcomes. Patient-specific implant selection and programmed endogenous protein exchange remain hypotheses until prospective validation shows added clinical value.

## Glossary

AN: non-bone animal evidence
BA: bone-or implant-relevant animal evidence
BMP: bone morphogenetic protein
CL: prospective clinical-outcome evidence
CQA: critical quality attribute
DIA: data-independent acquisition
ECM: extracellular matrix
EX: *ex vivo* human-biofluid evidence
FAK: focal adhesion kinase
Fc: fragment crystallizable
FGF: fibroblast growth factor
FN: fibronectin
HDX-MS: hydrogen-deuterium exchange mass spectrometry
HU: human tissue/*in vivo*/observational evidence
IV: *in vitro* defined-system evidence
LC-MS/MS: liquid chromatography-tandem mass spectrometry
MSC: mesenchymal stromal cell
NET: neutrophil extracellular trap
PAD4: peptidyl arginine deiminase 4
PDGF: platelet-derived growth factor
PEA: poly(ethyl acrylate)
QCM-D: quartz crystal microbalance with dissipation monitoring
RGD: Arg-Gly-Asp
SPR: surface plasmon resonance
TGF-β: transforming growth factor beta
ToF-SIMS: time-of-flight secondary ion mass spectrometry
VEGF: vascular endothelial growth factor
AFM: atomic force microscopy
HSA: human serum albumin
PMA: poly(methyl acrylate)
PRP: platelet-rich plasma
PRF: platelet-rich fibrin
A-PRF: advanced platelet-rich fibrin
NIPAM/BAM: N-isopropylacrylamide/N-tert-butylacrylamide
